# Interactions of *Saccharomyces cerevisiae* and *Lactiplantibacillus plantarum* Isolated from Light-Flavor Jiupei at Various Fermentation Temperatures

**DOI:** 10.3390/foods13182884

**Published:** 2024-09-12

**Authors:** Pu Yang, Bo Xi, Ying Han, Jiayang Li, Lujun Luo, Chaofan Qu, Junfang Li, Shuai Liu, Le Kang, Baoqing Bai, Ben Zhang, Shaojie Zhao, Pan Zhen, Lizhen Zhang

**Affiliations:** 1 School of Xinghuacun, Shanxi University, Taiyuan 030006, China; yangpu@sxu.edu.cn (P.Y.); baoqingbai@sxu.edu.cn (B.B.); benzhang@sxu.edu.cn (B.Z.); zhaoshaojie@sxu.edu.cn (S.Z.); 2 Shanxi Province Key Lab. of Plant Extraction and Health of Lujiu, Shanxi Xinghuacun Fenjiu Distillery Co., Ltd., Lvliang 032205, Chinaluolujun56@163.com (L.L.);; 3 School of Life Science, Shanxi University, Taiyuan 030006, China; 4 Institute of Biotechnology, Shanxi University, Taiyuan 030006, China; 5 Shanxi Province Science and Technology Resources and Large-Scale Instrument Open Sharing Center, Taiyuan 030000, China

**Keywords:** *Saccharomyces cerevisiae*, *Lactiplantibacillus plantarum*, interaction, Chinese Baijiu

## Abstract

Chinese Baijiu is a famous fermented alcoholic beverage in China. Interactions between key microorganisms, i.e., *Saccharomyces cerevisiae* and *Lactiplantibacillus plantarum*, have recently been reported at specific temperatures. However, empirical evidence of their interactions at various temperatures during fermentation is lacking. The results of this study demonstrated that *S. cerevisiae* significantly suppressed the viability and lactic acid yield of *L. plantarum* when they were cocultured above 15 °C. On the other hand, *L. plantarum* had no pronounced effect on the growth and ethanol yield of *S. cerevisiae* in coculture systems. *S. cerevisiae* was the main reducing sugar consumer. Inhibition of lactic acid production was also observed when elevated cell density of *L. plantarum* was introduced into the coculture system. A proteomic analysis indicated that the enzymes involved in glycolysis, lactate dehydrogenase, and proteins related to phosphoribosyl diphosphate, ribosome, and aminoacyl-tRNA biosynthesis in *L. plantarum* were less abundant in the coculture system. Collectively, our data demonstrated the antagonistic effect of *S. cerevisiae* on *L. plantarum* and provided insights for effective process management in light-flavor Baijiu fermentation.

## 1. Introduction

Chinese Baijiu is a traditional popular distilled liquor in China. Chinese Baijiu brewing is a solid-state fermentation process. Jiupei refers to fermented grains with single or various grains used as the substrate and with Jiuqu as a starter [1,2]. Chinese Baijiu contains rich flavor compounds. Water and ethanol are commonly present in all types of Chinese Baijiu, while the trace components, including alcohols, esters, ketones, phenols, terpenes, aldehydes, acids, and sulfur compounds, are different between types and grades [3,4]. Although they are at low concentrations, the profile of these trace components is critical to the flavor of a particular Baijiu [5].

Microorganisms are the main contributors to the biosynthesis of these flavor compounds [6]. In addition to Jiuqu, the microbiota involved in Chinese Baijiu fermentation also originate from the surrounding environment, including the surfaces of the jars, the outdoor and indoor ground, the tools used, and air [6,7,8]. The substrate in the grains, such as starch, can be fermented to ethanol and other flavor compounds via microbial metabolic activities. The microbial community structure in Chinese Baijiu fermentation changes throughout the fermentation process. In light-flavor Baijiu fermentation, various microorganisms are present in the initial stage of fermentation, while *Saccharomyces* and *Lactobacillus* are the dominant fungal and bacterial genera after seven days of fermentation [7]. Among them, *Saccharomyces cerevisiae* is the main contributor of ethanol, which accounts for 65–67% (*v*/*v*) in distilled products. *S. cerevisiae* also produces other flavor components, such as higher alcohols [9], 2-furfurylthiol [10], and benzenemethanethiol [11]. *Lactiplantibacillus plantarum* (previously *Lactobacillus plantarum*) is a key lactic acid contributor [12,13]. Lactic acid, the major organic acid in light-flavor Baijiu fermentation [14], is the precursor of ethyl lactate, a key flavor component in light-flavor Baijiu. However, the accumulation of lactic acid drives the succession of, as well as a functional shift in, the microbiota in Jiupei [15]. Thus, *S. cerevisiae* and *L. plantarum* are among the most important species in light-flavor Baijiu fermentation.

Diverse interactions are found between *S. cerevisiae* and *L. plantarum*. Though co-inoculation with *L. plantarum* could improve the ethanol tolerance of *S. cerevisiae* [16,17], growth inhibition of *S. cerevisiae* by *L. plantarum* is also reported [16,18]. In contrast, an extended lag phase, reduced biomass, and lower lactic acid yield of *L. plantarum* were found in coculture with *S. cerevisiae* [19]. Additionally, Gerardi et al. found diverse interactions between *L. plantarum* strains and *S. cerevisiae*. *S. cerevisiae* hampered the growth of *L. plantarum* FG61, FG68, and FG69 while enhancing the growth of *L. plantarum* CI180-11 [20]. Thus, the interactions between *S. cerevisiae* and *L. plantarum* may impact the yield and flavor composition in Baijiu fermentation.

Environmental factors, such as acidity, moisture, and temperature, also influence microbial community succession and flavor composition during fermentation [21]. Among them, temperature is a key parameter in Chinese Baijiu fermentation. A higher fermentation temperature (37 °C vs. 28 °C) enhances *Lactobacillus* growth and hinders *Saccharomyces* growth [21]. Additionally, the esterase activities of *Lactobacillus* species are affected by temperature [22]. Interestingly, *B. licheniformis* inhibits the growth rate of *Zygosaccharomyces bailii* at 37 °C but not at 30 °C [23]. In light-flavor Baijiu fermentation, the temperature of Jiupei starts as low as 9 °C in the initial stage, progressively increasing to 30 °C on day 7, and then slowly decreasing to around 24 °C at the end of fermentation. However, how fermentation temperature influences the interactions between *S. cerevisiae* and *L. plantarum* remains unclear.

In this study, the interactions between *S. cerevisiae* Y28 and *L. plantarum* R2 were investigated by inoculating these strains in monoculture and coculture systems, respectively. Both strains were isolated from light-flavor Jiupei in a previous study [13]. The former was a key ethanol contributor, and the latter was a key lactic acid producer [13]. Then, the microbial biomass, pH, and concentration of reducing sugars, as well as the key metabolites, i.e., lactic acid and ethanol, were characterized. Additionally, a proteomic analysis was applied to investigate the response of *L. plantarum* to the presence of *S. cerevisiae*. Our study will provide valuable insights for effective process management in Chinese Baijiu fermentation.

## 2. Materials and Methods

### 2.1. Strains and Cultural Medium

*S. cerevisiae* Y28 and *L. plantarum* R2 were isolated from Chinese light-flavor Baijiu Jiupei from Shanxi in a previous study [13].

Sorghum extract broth (SEB) was employed in the fermentation process. The preparation of SEB was as follows: sorghum was boiled in water (1:5, *m*/*v*) for 1 h. When it cooled down, the solution was treated with 120 U/mL α-amylase (Beijing Solarbio Science & Technology Co., Ltd., Beijing, China) and 600 U/mL amyloglucosidase (Solarbio) until the starch was completely degraded, which was determined using an iodine solution (5%). Then, the medium was incubated at 90 °C for 30 min, filtered using gauze, and centrifuged at 12,000× *g* at 25 °C for 10 min to collect the supernatant. Finally, the medium was adjusted to pH 5.5 with 1 mol/L HCl and autoclaved at 115 °C for 30 min before use.

### 2.2. Cultural Conditions and Growth Monitoring

*S. cerevisiae* Y28 and *L. plantarum* R2 were cultured for 24 h in SEB at 28 °C and 35 °C, respectively, and transferred into fresh SEB at 1:1 to obtain the coculture system. The initial biomass was 10^6^ CFU/mL. The *S. cerevisiae* Y28 and *L. plantarum* R2 monocultures were used as controls. The cultures were incubated statically at 30, 27, 24, 21, 18, 15, 12, and 9 °C and were routinely sampled at 0 h, 6 h, 12 h, 24 h, day 2, day 4, day 7, and day 10. To monitor microbial growth, the culture was serially diluted and spread on yeast extract peptone dextrose agar for colony counting. *S. cerevisiae* Y28 and *L. plantarum* R2 were distinguished by colony morphology (Figure S1). Then, the culture was centrifuged, and the supernatant was subjected to pH measurement with a pH meter (INESA Scientific Instrument Co., Ltd., Shanghai, China).

### 2.3. Lactic Acid, Ethanol, and Reducing Sugar Measurement

Ethanol and lactic acid in the supernatant were determined using gas chromatography (GC) and high-performance liquid chromatography (HPLC), respectively.

GC was performed using an Agilent 7820 A VL GC System (Agilent, Santa Clara, USA) equipped with an Agilent G3903-63008 column (30 m, 0.25 nm, 0.25 μm) and flame ionization detector. The initial temperature of the column was 40 °C for 1 min. The temperature of the column was increased to 150 °C at 5 °C/min and then to 210 °C at 15 °C/min. Finally, the column was kept at 210 °C for 3 min. The injection volume for GC was 1 μL.

A Waters e2695 (Waters, Milford, MA, USA) coupled with a Supersil AQ-C18 column (4.6 mm × 250 mm, 5 μm, Dalian Elite Analytical Instruments Co., Ltd., Dalian, China) and a UV detector at 215 nm was employed for HPLC. The temperature of the column was kept at 35 °C. KH_2_PO_4_ (0.01 mol/L, pH 2.88) was used as a mobile phase at a flow rate of 0.5 mL/min. The injection volume for HPLC was 10 μL.

Reducing sugars were determined using a Reducing Sugar Content Assay Kit (Solarbio) according to the manufacturer’s protocol.

### 2.4. Proteomic Analysis

To perform proteomic analysis, the cells inoculated at 30 °C were collected at 14 h and 16 h via centrifugation. The cells were resuspended in lysis buffer (1%SDS, 8 mol/L urea) containing a protease inhibitor cocktail (Bimake, Houston, TX, USA) and treated using an MP Fastprep-24 5G tissue homogenizer (MP Biomedicals, Santa Ana, CA, USA). The samples were incubated on ice for 30 min and vortexed for 5 s every 5 min. The samples were then centrifuged at 12,000× *g* for 20 min, and the supernatant was collected. The protein in the supernatant was checked with SDS-PAGE and quantified using a Pierce™ BCA Protein Assay Kit (ThermoFisher Scientific, Waltham, MA, USA).

One hundred micrograms of protein was mixed with lysis buffer and triethylammonium bicarbonate buffer (TEAB) to obtain 1 mg/mL protein and 100 mM TEAB. The protein solution was treated with 10 mM Tris-(2-carboxyethyl)-phosphine for 60 min at 37 °C and then treated with 40 mM iodoacetamide for 40 min at room temperature in darkness. The reaction mixture was then treated with precooled acetone (1:6, *v*/*v*) for 4 h at −20 °C and centrifuged at 12,000× *g* for 20 min. The protein pellet was resuspended in 100 mM TEAB and digested by trypsin at 37 °C overnight.

After trypsin digestion, the peptides were dried with an LNG-T98 vacuum pump (Huamei Biochemical Instrument Factory, Taicang, China) and resuspended in 8 μL 0.1% trifluoroacetic acid (*v*/*v*). Then, the peptides were desalted using an Oasis HLB 96-well plate (Waters, Milford, MA, USA), dried using an LNG-T98 vacuum pump, dissolved in water, and quantified using a Pierce Quantitative Colorimetric Peptide Assay (ThermoFisher Scientific).

The generated peptides were analyzed using a VanquishNeo UHPLC (ThermoFisher Scientific) equipped with an ES906 column (150 μm × 15 cm, ThermoFisher Scientific). Solvent A (2% acetonitrile and 0.1% formic acid in water) and Solvent B (80% acetonitrile and 0.1% formic acid in water) were used as a mobile phase with the 180 SPD method. The flow rate of the mobile phase was 500 nL/min. MS data with an *m/z* range of 100 to 1700 were collected using an Orbitrap Astral mass spectrometer in DIA mode. The MS raw data were analyzed using Spectronaut™ 18 against *Lactobacillus plantarum* (Taxon ID 1590, https://www.uniprot.org/uniprot, accessed on 30 August 2024) with the following parameters: Protein FDR ≤ 0.01, Peptide FDR ≤ 0.01, Peptide Confidence ≥ 99%, and XIC width ≤ 75 ppm. Finally, the data were submitted to the Kyoto Encyclopedia of Genes and Genomes (KEGG) (https://www.kegg.jp, accessed on 17 October 2023) to analyze metabolic pathways.

### 2.5. Statistical Analysis

All experiments were performed in triplicate. The data are presented as the mean ± standard deviation. Statistical significance between two groups was determined using *t*-test, while comparisons among three groups were conducted using one-way analysis of variance. *p* < 0.05 was used as the criteria for significant differences. The proteomic data were analyzed using Majorbio Cloud Platform [24]. Differentially expressed proteins were defined with the following criteria: |Log_2_FC| > 1 and *p* < 0.05.

## 3. Results

### 3.1. Microbial Biomass in Monoculture and Coculture Systems

**Figure 1:**
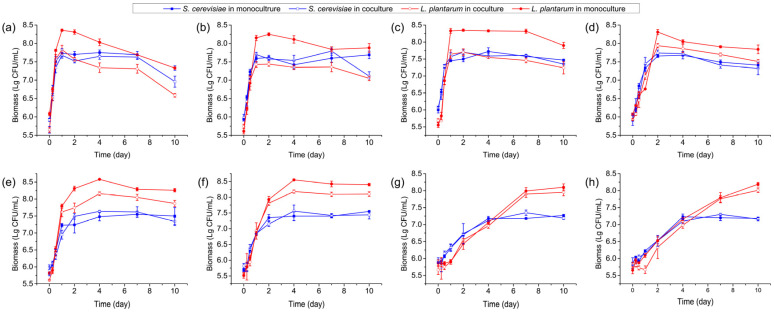
Microbial biomass in monoculture and coculture systems at various temperatures: (**a**) 30 °C; (**b**) 27 °C; (**c**) 24 °C; (**d**) 21 °C; (**e**) 18 °C; (**f**) 15 °C; (**g**) 12 °C; and (**h**) 9 °C.

*S. cerevisiae* Y28 growth occurred at all inoculated temperatures. It reached the stationary phase at 24 h when cultured at 24–30 °C. However, it took 48 h to reach the stationary phase when *S. cerevisiae* Y28 was cultured at 15–21 °C, while 4 days were needed to reach the stationary phase when *S. cerevisiae* Y28 was cultured at 12 and 9 °C. The biomass of *S. cerevisiae* Y28 at the stationary phase was higher than 10^7^ CFU/mL when it was incubated above 15 °C (Figure 1).

A similar trend was found when *L. plantarum* R2 was inoculated in the monoculture. An extended exponential phase was observed at lower temperatures, while the highest cell densities were above 10^8^ CFU/mL at all temperatures. However, after a period of rapid growth in the first 24 h, the biomass of *L. plantarum* R2 dropped significantly, from 10^8.36±0.03^ CFU/mL at day 1 to 10^7.33±0.03^ at day 10 at 30 °C (Figure 1a).

*L. plantarum* R2 had little effect on the growth rate of *S. cerevisiae* Y28 in the coculture system (Figure 1, Table S1). However, the biomass of *S. cerevisiae* Y28 in the coculture system significantly decreased from 10^7.63±0.07^ CFU/mL and 10^7.79±0.11^ CFU/mL at day 7 to 10^6.96±0.15^ CFU/mL and 10^7.11±0.12^ CFU/mL at day 10, when inoculated at 30 °C and 27 °C, respectively. In contrast, when incubated at 30 °C, the biomass of *L. plantarum* R2 reached 10^8.36±0.03^ CFU/mL in the monoculture system at day 1, while the biomass was 10^7.83±0.12^ CFU/mL in the coculture system (Figure 1). Similarly, when the temperature was above 15 °C, the population sizes of *L. plantarum* R2 in the coculture systems at the stationary phase were significantly lower than those in the monoculture systems, albeit with low significance at several sampling times (Table S1).

### 3.2. pH Changes in Monoculture and Coculture Systems

**Figure 2:**
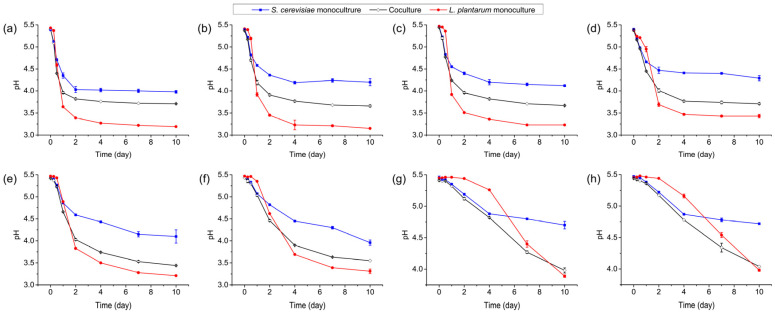
pH changes in monoculture and coculture systems at various temperatures: (**a**) 30 °C; (**b**) 27 °C; (**c**) 24 °C; (**d**) 21 °C; (**e**) 18 °C; (**f**) 15 °C; (**g**) 12 °C; and (**h**) 9 °C.

Throughout the incubation period, the pH of the *S. cerevisiae* Y28 monoculture and *L. plantarum* R2 monoculture exhibited a downward trend (Figure 2), indicating the biosynthesis of organic acid by these strains. Additionally, the pH reduction rate slowed at lower temperatures, demonstrating that lower temperatures restrained the organic acid fermentation of *S. cerevisiae* Y28 and *L. plantarum* R2. In the coculture system, the pH of the culture was significantly lower than that in the *S. cerevisiae* Y28 monoculture and was significantly higher than that in the *L. plantarum* R2 monoculture after day 1, day 2, and day 4, when it was incubated at 24–30 °C, 18–21 °C, and 15 °C, respectively (Table S2), suggesting that the organic acid yield of *L. plantarum* R2 was hampered by *S. cerevisiae* Y28.

### 3.3. Lactic Acid Yield of L. plantarum R2 in Monoculture and Coculture Systems

**Figure 3:**
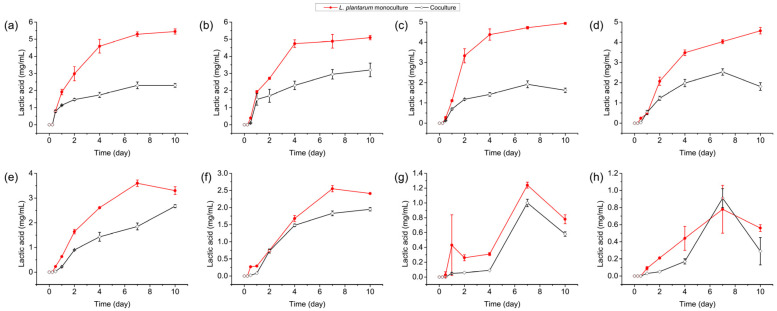
Lactic acid yield of *Lactiplantibacillus plantarum* R2 in monoculture and coculture systems at various temperatures: (**a**) 30 °C; (**b**) 27 °C; (**c**) 24 °C; (**d**) 21 °C; (**e**) 18 °C; (**f**) 15 °C; (**g**) 12 °C; and (**h**) 9 °C.

Lactic acid was not detected in the *S. cerevisiae* Y28 monoculture; thus, we focused on the culture in the presence of *L. plantarum* R2. In the *L. plantarum* R2 monoculture system, the concentration of lactic acid increased during incubation, whereas the yield of lactic acid decreased at lower temperatures (Figure 3). The concentrations of lactic acid increased to 5.45 ± 0.15 mg/mL, 5.09 ± 0.13 mg/mL, 4.94 ± 0.06 mg/mL, 4.03 ± 0.10 mg/mL, and 3.30 ± 0.16 mg/mL at day 10, when it was inoculated from 30 to 18 °C. On the other hand, when *L. plantarum* R2 was inoculated at temperatures below 15 °C, the highest concentrations of lactic acid were observed at day 7, with values of 2.55 ± 0.09 mg/mL (15 °C), 1.24 ± 0.04 mg/mL (12 °C), and 0.78 ± 0.28 mg/mL (9 °C); these decreased to 2.41 ± 0.02 mg/mL (15 °C), 0.78 ± 0.06 mg/mL (12 °C), and 0.56 ± 0.04 mg/mL (9 °C) at day 10.

As expected, when *L. plantarum* R2 was inoculated with *S. cerevisiae* Y28 at 30 °C, the lactic acid yield was 2.30 ± 0.11 mg/mL at day 10, which was significantly lower than that in the monoculture. The inhibitory effect on lactic acid fermentation by *S. cerevisiae* Y28 was also observed when it was cultured above 15 °C (Figure 3 and Table S3), which was consistent with the pH differences between the *L. plantarum* R2 monoculture and the coculture systems.

### 3.4. Ethanol Production by S. cerevisiae Y28 in Monoculture and Coculture Systems

**Figure 4:**
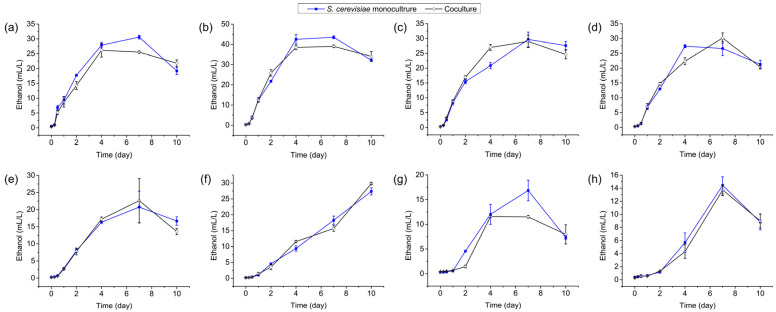
Ethanol production by *Saccharomyces cerevisiae* Y28 in monoculture and coculture systems at various temperatures: (**a**) 30 °C; (**b**) 27 °C; (**c**) 24 °C; (**d**) 21 °C; (**e**) 18 °C; (**f**) 15 °C; (**g**) 12 °C; and (**h**) 9 °C.

*L. plantarum* R2 did not produce ethanol; thus, we focused on the culture with *S. cerevisiae* Y28 inoculation. In the *S. cerevisiae* Y28 monoculture system, the concentration of ethanol in the culture increased (Figure 4). However, the concentration of ethanol decreased from day 7 to day 10 when *S. cerevisiae* Y28 was incubated above 18 °C. When *S. cerevisiae* Y28 was inoculated at 15 °C, ethanol was constantly accumulated until day 10. Compared to those at higher inoculation temperatures, the yields of ethanol significantly decreased when *S. cerevisiae* Y28 was inoculated at 12 °C and 9 °C, with values of 16.84 ± 2.09 mL/L (12 °C) and 14.44 ± 1.31 mL/L (9 °C) at day 7. When *S. cerevisiae* Y28 was inoculated with *L. plantarum* R2, the ethanol yield was comparable to that in the monoculture system regardless of the incubation temperature (Table S4).

### 3.5. Consumption of Reducing Sugars

**Figure 5:**
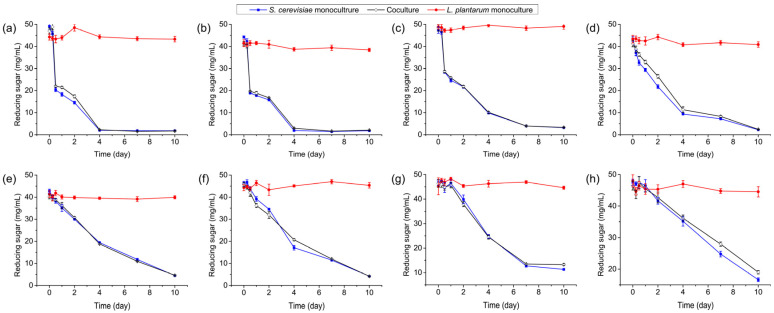
Utilization of reducing sugars in monoculture and coculture systems at various temperatures: (**a**) 30 °C; (**b**) 27 °C; (**c**) 24 °C; (**d**) 21 °C; (**e**) 18 °C; (**f**) 15 °C; (**g**) 12 °C; and (**h**) 9 °C.

As shown in Figure 5, when the *S. cerevisiae* Y28 monoculture was cultured at temperatures above 24 °C, the consumption of reducing sugars was divided into two phases. In the first phase, 28.80 mg/mL, 25.37 mg/mL, and 18.90 mg/mL of reducing sugars were utilized in the first 12 h when *S. cerevisiae* Y28 was inoculated at 30 °C, 27 °C, and 24 °C, respectively. In the second phase, the concentration of reducing sugars dropped to less than 2 mg/mL at day 4, when *S. cerevisiae* Y28 was inoculated at 30 °C and 27 °C, while the concentration of reducing sugars reached 3.95 ± 0.18 mg/mL at day 7, when *S. cerevisiae* Y28 was inoculated at 24 °C. On the other hand, only one phase was detected when *S. cerevisiae* Y28 was inoculated at 21 °C and 18 °C, and the concentration of reducing sugars continued to decrease during the culture process. When *S. cerevisiae* Y28 was inoculated at 15–9 °C, the concentration of reducing sugars remained steady for 6 h at 15 °C and for 24 h at 12 °C and 9 °C before declining until day 10.

When *L. plantarum* R2 was inoculated in the monoculture, the concentration of reducing sugars remained unchanged from day 0 to day 10 (Figure 5).

In the coculture system, the consumption of reducing sugars was in a manner similar to that in the *S. cerevisiae* Y28 monoculture system, regardless of the presence of *L. plantarum* R2 (Figure 5 and Table S5).

### 3.6. Effect of Elevated Initial Biomass of L. plantarum R2 on Interactions

**Figure 6:**
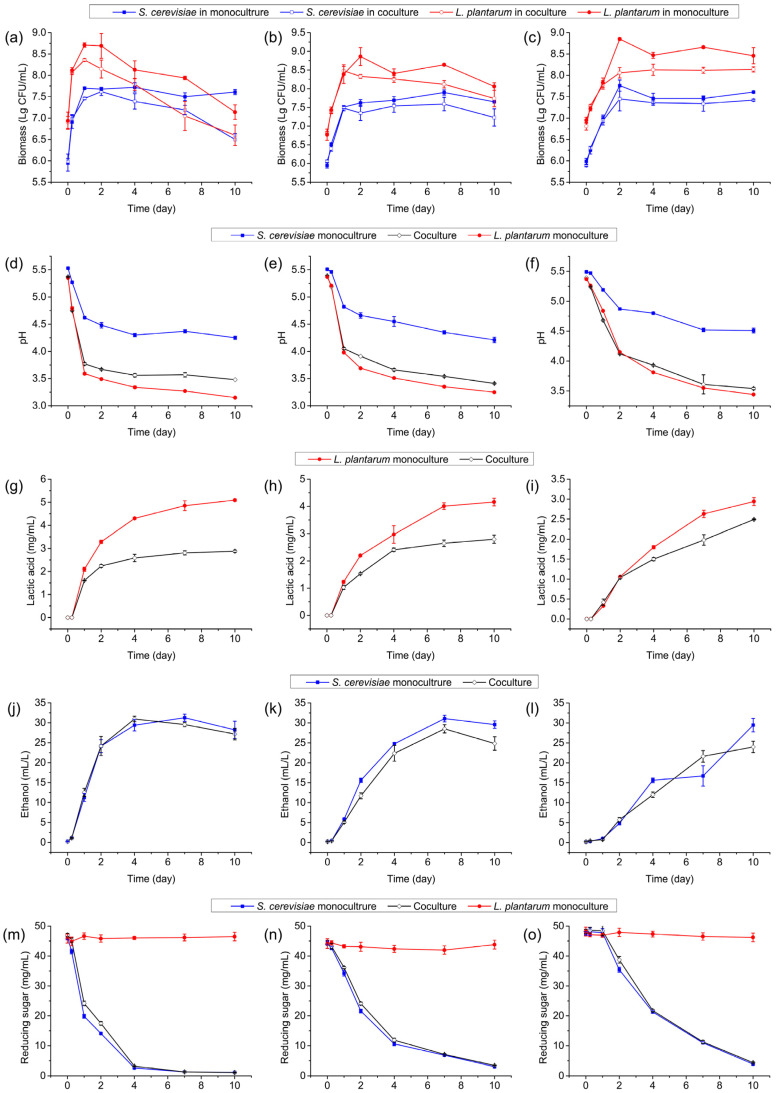
Microbial biomass (**a**–**c**), pH changes (**d**–**f**), lactic acid yield (**g**–**i**), ethanol yield (**j**–**l**), and utilization of reducing sugars (**m**–**o**) in monoculture and coculture systems under 30 °C (**a**,**d**,**g**,**j**,**m**), 21 °C (**b**,**e**,**h**,**k**,**n**), and 15 °C (**c**,**f**,**i**,**l**,**o**).

To investigate whether the inhibitory effect could be alleviated, we increased the initial biomass of *L. plantarum* R2 to 10^7^ CFU/mL, obtaining an initial biomass ratio of *S. cerevisiae* Y28 to *L. plantarum* R2 of 1:10. The population density of *L. plantarum* R2 in the coculture system was lower, albeit with low significance, compared to that in the monoculture system at all three incubation temperatures (Figure 6a–c and Table S1), indicating an inhibitory effect of *S. cerevisiae* Y28 on *L. plantarum* R2, although the initial biomass of *L. plantarum* R2 was elevated. On the other hand, the cell density of *S. cerevisiae* Y28 in the coculture system was lower than that in the *S. cerevisiae* Y28 monoculture system, with low significance from day 4, when it was cultured at 30 °C (Figure 6a).

The pH of the coculture systems was significantly higher than that of the *L. plantarum* R2 monoculture at 30 °C and 21 °C (Figure 6d,e and Table S2), demonstrating the inhibitory effect of *S. cerevisiae* Y28 on lactic acid production by *L. plantarum* R2. The HPLC results confirmed this inhibitory effect (Figure 6g,h and Table S3). When the coculture system was inoculated at 15 °C, its pH was slightly, but insignificantly, higher than that in the *L. plantarum* R2 monoculture system at day 10 (Figure 6f), while the concentration of lactic acid in the coculture system was significantly lower than that in the *L. plantarum* R2 monoculture system (Figure 6i and Table S3).

The accumulation of ethanol was comparable between the *S. cerevisiae* Y28 monoculture system and the coculture system when it was inoculated at all three temperatures (Figure 6j–l). No differences were found between the *S. cerevisiae* Y28 monoculture and the coculture systems in terms of the consumption of reducing sugars (Figure 6m–o).

Thus, with an increase in the initial biomass of *L. plantarum* R2, inhibition of lactic acid production by *S. cerevisiae* Y28 was also observed, while the suppression of bacterial growth was alleviated.

### 3.7. Response of L. plantarum R2 to S. cerevisiae Y28 at Proteomic Level

To determine the sampling time for proteomic analysis, the growth curve of *L. plantarum* R2 in the monoculture and the coculture systems was measured. The results showed that the biomass of *L. plantarum* R2 in the coculture system was comparable to that in the monoculture system until 12 h post inoculation (hpi, Figure S2). However, *L. plantarum* R2 in the coculture system was in a stationary phase from 14 hpi, while *L. plantarum* R2 in the monoculture system was still in an exponential phase. Additionally, the biomass of *L. plantarum* R2 in the coculture system was significantly lower than that in the monoculture system from 14 hpi, indicating the significant inhibitory effect of *S. cerevisiae* Y28 on the growth of *L. plantarum* R2 from 14 hpi (Figure S2).

**Figure 7:**
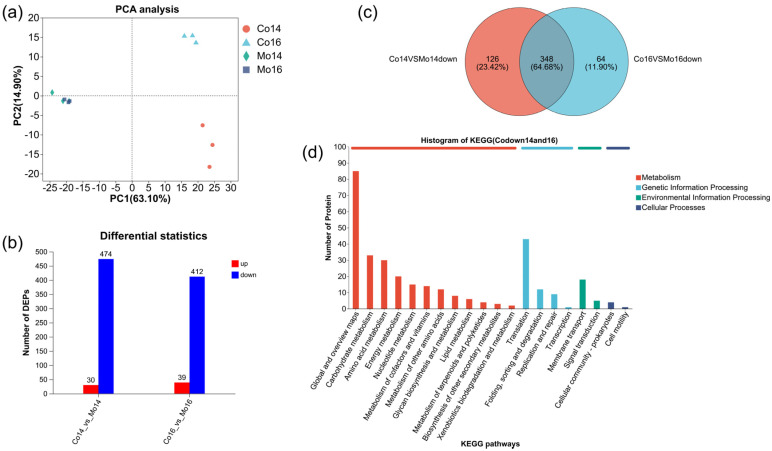
General features of the proteomic pattern of *L. plantarum*. (**a**) PCA analysis of the protein expression patterns of *L. plantarum* in the presence or absence of *S. cerevisiae*. (**b**) Number of differentially expressed proteins. (**c**) Venn map of the downregulated proteins in the coculture system between 14 h and 16 h. (**d**) KEGG pathways of the downregulated proteins in the coculture system shared at 14 h and 16 h. Co14, sampled at 14 h in the coculture system; Co16, sampled at 16 h in the coculture system; Mo14, sampled at 16 h in the monoculture system; and Mo16, sampled at 16 h in the monoculture system.

**Figure 8:**
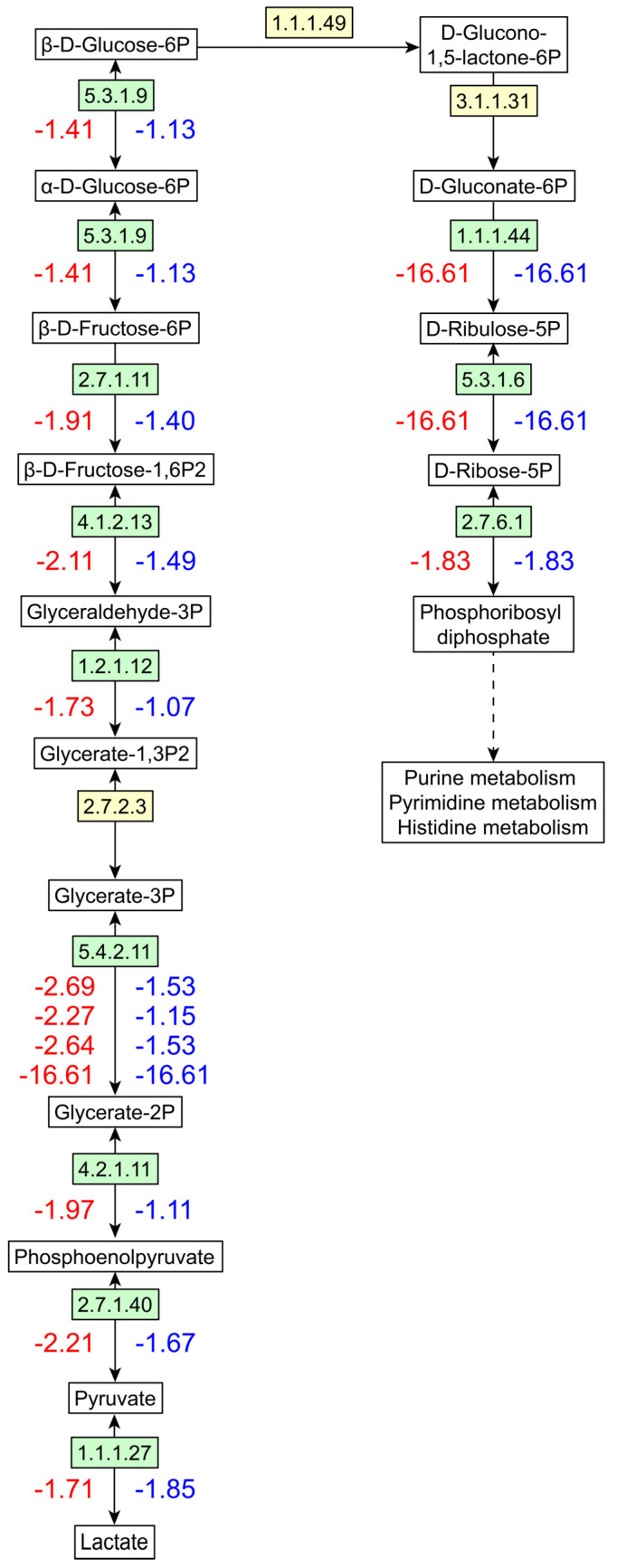
Changes in expression levels of proteins associated with lactic acid fermentation and phosphoribosyl diphosphate biosynthesis pathways.

To investigate the response of *L. plantarum* R2 to *S. cerevisiae* Y28 in the coculture system, cells were collected via centrifugation at 14 hpi and 16 hpi. A total of 740 *L. plantarum* proteins were identified using LC-MS/MS, with 725 proteins annotated using KEGG. A principal component analysis (PCA) indicated a clear discrimination of protein expression patterns between the monoculture and coculture systems, while the protein expression patterns of *L. plantarum* in the monoculture at 14 hpi and 16 hpi were similar (Figure 7a). At 14 hpi, 30 *L. plantarum* proteins were significantly upregulated and 474 were significantly downregulated in the coculture system (Figure 7b). Similarly, at 16 hpi, 39 *L. plantarum* proteins were significantly upregulated and 412 were significantly downregulated (Figure 7b). A total of 384 *L. plantarum* proteins showed lower abundance in the coculture system at both sampling time points (Figure 7c). Among them, 43 proteins belonged to translation, 33 proteins were assigned to carbohydrate metabolism, and 30 proteins were amino acid metabolism-associated proteins (Figure 7d). Further analysis indicated that 15 proteins were associated with glycolysis and part of the HMP pathway, as well as lactate dehydrogenase (Figure 8, Table S6). For translation, 34 proteins were related to ribosomes (Table S7), and 9 were aminoacyl-tRNA synthetases (Table S8).

## 4. Discussion

Diverse interactions between *S. cerevisiae* and *L. plantarum* have been reported in previous studies. In this study, the growth of *L. plantarum* R2 was inhibited by *S. cerevisiae* Y28, which is consistent with previous results [19,20]. In the coculture system, the growth of *L. plantarum* ceased at 14 h, which was an earlier finish than that in the monoculture system (Figure S2). An inhibitory effect was also found, though the initial biomass of *L. plantarum* was elevated (Figure 6). A PCA of proteomic data showed the differences in the protein expression patterns of *L. plantarum* between the coculture and monoculture systems, demonstrating the influence of *S. cerevisiae* on the activity of *L. plantarum*. A further analysis showed that 474 and 412 proteins were less abundant in the coculture system, while only 30 and 39 proteins were more abundant in the coculture system at 14 hpi and 16 hpi, respectively, indicating that the activity of *L. plantarum* was suppressed by *S. cerevisiae*. KEGG annotation indicated that the growth arrest of *L. plantarum* may be attributed to lower nucleotide, cofactor, and protein biosynthesis rates. D-ribose-5P and phosphoribosyl diphosphate (PRPP) are essential for the biosynthesis of purine and pyrimidine, which not only are utilized in DNA/RNA biosynthesis but also act as precursors of energy-carrying molecules, such as ATP and GTP. PRPP is also required in amino acid and cofactor biosynthesis [25]. In this study, a lower abundance of PRPP synthetase (EC 2.7.6.1) led to a lower concentration of PRPP, as well as a lower abundance of the products in the downstream pathways, i.e., nucleotides, amino acids, and cofactors. The lower biosynthesis efficiency of these essential molecules was one of the reasons for the growth arrest of *L. plantarum*. Ribosomes are the engine of protein synthesis and are essential for free-living organisms [26]. The ribosomal proteins were stable during constant cell growth and were degraded in the transition from the exponential phase to the stationary phase [27]. Previous studies have demonstrated that ribosome abundance is positively connected to the bacterial growth rate [28,29]. Thus, ribosome deficiency was another reason for the growth arrest of *L. plantarum* in the coculture system. Aminoacyl-tRNA is responsible for delivering amino acids to growing polypeptide chains with high fidelity [30]. The limitation of aminoacyl-tRNA results in growth inhibition in bacteria [31,32,33]. Thus, in the presence of *S. cerevisiae*, a lower abundance of proteins related to PRPP, ribosome, and aminoacyl-tRNA biosynthesis in *L. plantarum* (Figure 8, Tables S6–S8) resulted in lower efficiency of energy supply and DNA/RNA/cofactor/protein biosynthesis, leading to a lower growth rate.

The production of lactic acid was observed in the stationary phase in the monoculture (Figure 3). However, the lactic acid yield of *L. plantarum* R2 was suppressed in the coculture system (Figure 3). Lactic acid is the most abundant organic acid in light-flavor Baijiu Jiupei [14], while a high concentration of lactic acid is detrimental to *S. cerevisiae* [34,35], which is a key ethanol contributor. The boiling point of lactic acid is 122 °C, and it cannot be removed during distillation. The accumulation of lactic acid in Jiupei exerts a greater negative impact on the second round of light-flavor Baijiu fermentation. Thus, the control of the lactic acid yield via bioaugmented inoculation with *S. cerevisiae* is a promising method for increasing the ethanol yield in light-flavor Baijiu fermentation. The proteomic data indicated that enzymes related to glycolysis and lactate dehydrogenase were downregulated, which explained the inhibition of lactic acid fermentation. Lactic acid fermentation via glycolysis is also the primary energy source for *L. plantarum* in anaerobic conditions. Thus, a lower energy yield, owing to a lower level of lactic acid fermentation in the coculture, also resulted in *L. plantarum* growth arrest.

Reducing sugars are primary carbon sources for microbial growth, as well as for ethanol and lactic acid fermentation. Thus, the dynamics of reducing sugars are considered to be a useful indicator for evaluating the substrate utilization efficiency and fermentation process [36,37]. In order to give an overview of available carbon sources, in this study, reducing sugars instead of specific sugars were determined. In the presence of *S. cerevisiae*, the concentration of reducing sugars decreased alongside cell proliferation, as well as organic acid and ethanol fermentation. However, the reducing sugars did not decrease in the *L. plantarum* monoculture, even though population growth and lactic acid accumulation were observed. A similar result has also been reported in previous studies. Gerardi et al. found that the contents of glucose and fructose in *L. plantarum* inoculated culture were comparable to those in an uninoculated medium within 15 days of inoculation, though cell proliferation and organic acid production were present [20]. The reducing sugar content in *Betaphycus gelatinum* fermented with *Lactobacillus brevis* declines in the first 24 h and then increases [38]. This may be due to the hydrolase ability of *L. plantarum* on dextrin. Although the degradation of starch was indicated by an iodine solution, short-chain dextrin could not be detected by iodine [39]. During fermentation, the short-chain dextrin in the SEB could be degraded and consumed by *L. plantarum*. In this study, the consumption rate of reducing sugars and the degradation rate of short-chain dextrin by *L. plantarum* appeared to be similar, thus maintaining the concentration of reducing sugars during fermentation. Additionally, the consumption rates of reducing sugars in the *S. cerevisiae* monoculture and coculture systems were comparable, indicating that *S. cerevisiae* was the primary consumer of reducing sugars.

*S. cerevisiae* and *L. plantarum* entered the stationary phase in the presence of reducing sugars, which were then transferred to ethanol or lactic acid. Nutrient starvation is a key factor in the transition from the exponential to the stationary phase [40,41]. Since reducing sugars were not depleted in the culture, the limitation of other nutrients, such as nitrogen, phosphorus, and magnesium, may be the reason for microbial growth arrest. On the other hand, alcohol dehydrogenase [42] and lactate dehydrogenase [43] are observed in the stationary phase, suggesting that these enzymes may facilitate ethanol and lactic acid fermentation in the stationary phase.

Temperature is a key factor that influences the interactions between *S. cerevisiae* and other microorganisms. It was reported that a higher temperature enhances the competitiveness of *S. cerevisiae* against *Lachancea thermotolerans* [44,45]. A higher temperature also favors the growth of *S. cerevisiae* in the multi-species yeast consortium [46]. Additionally, the lactic acid yield in the *L. thermotolerans*–*S. cerevisiae* coculture system is higher at 20 °C than at 30 °C [44]. The growth inhibition of *S. cerevisiae* on *S. kudriavzevii* is also augmented at a higher fermentation temperature [47]. In this study, the inhibitory effect was enhanced at higher fermentation temperatures. Though a higher rate of reducing sugar consumption by *S. cerevisiae* has been found at higher temperatures [44], the reducing sugars were not exhausted in the early stationary phase. Thus, carbon source competition was not the primary reason for the inhibitory effect. How *S. cerevisiae* suppressed *L. plantarum* at high temperatures remains unknown.

In conclusion, our data demonstrated that a higher fermentation temperature enhanced the inhibitory effect of *S. cerevisiae* on the cell growth and lactic acid yield of *L. plantarum*, with its own growth and ethanol yield remaining unaffected. The inhibitory effect was attributed to the lower expression levels of glycolysis, ribosome, and aminoacyl-tRNA biosynthesis-associated proteins, as indicated by the proteomic analysis. Thus, fermentation temperature control is a key parameter in light-flavor Baijiu brewing. The role of *S. cerevisiae* in *L. plantarum* growth arrest in the coculture system at high temperatures should be addressed in the future.

## Data Availability

The proteomic raw data have been submitted to the National Genomic Data Center with accession number OMIX005891.

## Supplementary Materials

The following supporting information can be downloaded at https://www.mdpi.com/article/10.3390/foods13182884/s1: Figure S1: Colony morphology on yeast extract peptone dextrose agar. The larger colonies are *Saccharomyces cerevisiae*, while the smaller ones are *Lactiplantibacillus plantarum*; Figure S2: Growth curve of *L. plantarum* in the presence or absence of *S. cerevisiae*; Table S1: *T*-test of microbial biomass between monoculture and coculture systems; Table S2: One-way analysis of variance of pH between monoculture and coculture systems; Table S3: T-test of lactic acid yield between monoculture and coculture systems; Table S4: T-test of ethanol yield between monoculture and coculture systems; Table S5: One-way analysis of variance of reducing sugar between monoculture and coculture systems; Table S6: Changes in expression levels of proteins related to carbohydrate metabolism; Table S7: Changes in expression levels of proteins related to ribosome; Table S8: Changes in expression levels of proteins related to aminoacyl-tRNA biosynthesis.

## References

[1] Jin G., Zhu Y., Xu Y. (2017). Mystery behind Chinese liquor fermentation. Trends Biochem. Sci..

[2] Tu W., Cao X., Cheng J., Li L., Zhang T., Wu Q., Xiang P., Shen C., Li Q. (2022). Chinese Baijiu: The perfect works of microorganisms. Front. Microbiol..

[3] Li J., Zhang Q., Sun B. (2023). Chinese Baijiu and Whisky: Research reservoirs for flavor and functional food. Foods.

[4] Li H., Zhang X., Gao X., Shi X., Chen S., Xu Y., Tang K. (2023). Comparison of the aroma-active compounds and sensory characteristics of different grades of light-flavor Baijiu. Foods.

[5] Qiao L., Wang J., Wang R., Zhang N., Zheng F. (2023). A review on flavor of Baijiu and other world-renowned distilled liquors. Food Chem. X.

[6] Zhang P., Liu Y., Li H., Hui M., Pan C. (2024). Strategies and challenges of microbiota regulation in Baijiu brewing. Foods.

[7] Pang X., Han B., Huang X., Zhang X.Z., Hou L., Cao M., Gao L., Hu G., Chen J. (2018). Effect of the environment microbiota on the flavour of light-flavour Baijiu during spontaneous fermentation. Sci. Rep..

[8] Wang X., Du H., Zhang Y., Xu Y. (2018). Environmental microbiota drives microbial succession and metabolic profiles during Chinese liquor fermentation. Appl. Environ. Microbiol..

[9] Wei J., Du H., Xu Y. (2024). Revealing the key microorganisms producing higher alcohols and their assembly processes during Jiang-flavor Baijiu fermentation. Food Biosci..

[10] Zha M., Sun B., Yin S., Mehmood A., Cheng L., Wang C. (2018). Generation of 2-Furfurylthiol by carbon–sulfur lyase from the Baijiu yeast *Saccharomyces cerevisiae* G20. J. Agric. Food Chem..

[11] Zhang G., Xiao P., Xu Y., Li H., Li H., Sun J., Sun B. (2023). Isolation and characterization of yeast with benzenemethanethiol synthesis ability isolated from Baijiu Daqu. Foods.

[12] Zhao X., Li J., Du G., Chen J., Ren T., Wang J., Han Y., Zhen P., Zhao X. (2022). The influence of seasons on the composition of microbial communities and the content of lactic acid during the fermentation of fen-flavor Baijiu. Fermentation.

[13] Zhang X. (2022). Metabolic Activity Assessment of Key Microorganisms in Jiupei of Light-Flavor Baijiu. Master’s Thesis.

[14] Xue Y.A., Tang F., Cai W., Zhao X., Song W., Zhong J.A., Liu Z., Guo Z., Shan C. (2022). Bacterial diversity, organic acid, and flavor analysis of dacha and ercha fermented grains of fen flavor Baijiu. Front. Microbiol..

[15] Wei J., Nie Y., Du H., Xu Y. (2024). Reduced lactic acid strengthens microbial community stability and function during Jiang-flavour Baijiu fermentation. Food Biosci..

[16] Ding Y., Niu Y., Chen Z., Dong S., Li H. (2021). Discovery of novel *Lactobacillus plantarum* co-existence-associated influencing factor(s) on *Saccharomyces cerevisiae* fermentation performance. LWT Food Sci. Technol..

[17] He X., Liu B., Xu Y., Chen Z., Li H. (2021). Effects of *Lactobacillus plantarum* on the ethanol tolerance of *Saccharomyces cerevisiae*. Appl. Environ. Microbiol..

[18] Liu J., Huang T., Liu G., Ye Y., Soteyome T., Seneviratne G., Xiao G., Xu Z., Kjellerup B.V. (2022). Microbial interaction between *Lactiplantibacillus plantarum* and *Saccharomyces cerevisiae*: Transcriptome level mechanism of cell-cell antagonism. Microbiol. Spectr..

[19] Zhang Q., Sun Q., Tan X., Zhang S., Zeng L., Tang J., Xiang W. (2020). Characterization of γ-aminobutyric acid (GABA)-producing *Saccharomyces cerevisiae* and coculture with *Lactobacillus plantarum* for mulberry beverage brewing. J. Biosci. Bioeng..

[20] Gerardi C., Tristezza M., Giordano L., Rampino P., Perrotta C., Baruzzi F., Capozzi V., Mita G., Grieco F. (2019). Exploitation of *Prunus mahaleb* fruit by fermentation with selected strains of *Lactobacillus plantarum* and *Saccharomyces cerevisiae*. Food Microbiol..

[21] Zhang H., Wang L., Wang H., Yang F., Chen L., Hao F., Lv X., Du H., Xu Y. (2021). Effects of initial temperature on microbial community succession rate and volatile flavors during Baijiu fermentation process. Food Res. Int..

[22] Chen C., Xiong Y., Xie Y., Zhang H., Jiang K., Pang X., Huang M. (2022). Metabolic characteristics of lactic acid bacteria and interaction with yeast isolated from light-flavor Baijiu fermentation. Food Biosci..

[23] Zhuang X., Wu Q., Xu Y. (2017). Physiological characteristics of *Zygosaccharomyces bailii* and its interaction with *Bacillus licheniformis* in Chinese maotai-flavor liquor making. Microbiol. China.

[24] Ren Y., Yu G., Shi C., Liu L., Guo Q., Han C., Zhang D., Zhang L., Liu B., Gao H. (2022). Majorbio Cloud: A one-stop comprehensive bioinformatic platform for multiomics analyses. iMeta.

[25] Hove-Jensen B., Andersen K.R., Kilstrup M., Martinussen J., Switzer R.L., Willemoës M. (2016). Phosphoribosyl diphosphate (PRPP): Biosynthesis, enzymology, utilization, and metabolic significance. Microbiol. Mol. Biol. Rev..

[26] Bergkessel M. (2020). Regulation of protein biosynthetic activity during growth arrest. Curr. Opin. Microbiol..

[27] Piir K., Paier A., Liiv A., Tenson T., Maiväli Ü. (2011). Ribosome degradation in growing bacteria. EMBO Rep..

[28] Dai X., Zhu M. (2020). Coupling of ribosome synthesis and translational capacity with cell growth. Trends Biochem. Sci..

[29] Scott M., Gunderson C.W., Mateescu E.M., Zhang Z., Hwa T. (2010). Interdependence of cell growth and gene expression: Origins and consequences. Science.

[30] Hausmann C.D., Ibba M. (2008). Aminoacyl-tRNA synthetase complexes: Molecular multitasking revealed. FEMS Microbiol. Rev..

[31] Ferro I., Liebeton K., Ignatova Z. (2017). Growth-rate dependent regulation of tRNA level and charging in *Bacillus licheniformis*. J. Mol. Biol..

[32] Ho J.M., Bakkalbasi E., Soll D., Miller C.A. (2018). Drugging tRNA aminoacylation. RNA Biol..

[33] Zong B., Xiao Y., Li R., Li H., Wang P., Yang X., Zhang Y. (2023). Transcriptome and metabolome profiling to elucidate the mechanism underlying the poor growth of *Streptococcus suis* serotype 2 after orphan response regulator CovR deletion. Front. Vet. Sci..

[34] Deng N., Du H., Xu Y. (2020). Cooperative response of *Pichia kudriavzevii* and *Saccharomyces cerevisiae* to lactic acid stress in Baijiu fermentation. J. Agric. Food. Chem..

[35] Narendranath N.V., Thomas K.C., Ingledew W.M. (2001). Effects of acetic acid and lactic acid on the growth of *Saccharomyces cerevisiae* in a minimal medium. J. Ind. Microbiol. Biotechnol..

[36] Huang L.P., Jin B., Lant P. (2005). Direct fermentation of potato starch wastewater to lactic acid by *Rhizopus oryzae* and *Rhizopus arrhizus*. Bioprocess. Biosyst. Eng..

[37] Li J., Tang X., Qian H., Yang Y., Zhu X., Wu Q., Mu Y., Huang Z. (2021). Analysis of saccharification products of high-concentration glutinous rice fermentation by *Rhizopus nigricans* Q3 and alcoholic fermentation of *Saccharomyces cerevisiae* GY-1. ACS Omega.

[38] Wang Z., Zhao C., Guo Z., Li S., Zhu Z., Grimi N., Xiao J. (2023). Fermentation of *Betaphycus gelatinum* using *Lactobacillus brevis*: Growth of probiotics, total polyphenol content, polyphenol profile, and antioxidant capacity. Foods.

[39] Bailey J.M., Whelan W.J. (1961). Physical properties of starch: I. relationship between Iodine stain and chain length. J. Biol. Chem..

[40] Dworkin J., Harwood C.S. (2022). Metabolic reprogramming and longevity in quiescence. Annu. Rev. Microbiol..

[41] Breeden L.L., Tsukiyama T. (2022). Quiescence in *Saccharomyces cerevisiae*. Annu. Rev. Genet..

[42] den Ridder M., van den Brandeler W., Altiner M., Daran-Lapujade P., Pabst M. (2023). Proteome dynamics during transition from exponential to stationary phase under aerobic and anaerobic conditions in yeast. Mol. Cell. Proteom..

[43] Marco M.L., Kleerebezem M. (2008). Assessment of real-time RT-PCR for quantification of *Lactobacillus plantarum* gene expression during stationary phase and nutrient starvation. J. Appl. Microbiol..

[44] Gobbi M., Comitini F., Domizio P., Romani C., Lencioni L., Mannazzu I., Ciani M. (2013). *Lachancea thermotolerans* and *Saccharomyces cerevisiae* in simultaneous and sequential co-fermentation: A strategy to enhance acidity and improve the overall quality of wine. Food Microbiol..

[45] Joran A., Klein G., Roullier-Gall C., Alexandre H. (2022). Multiparametric approach to interactions between *Saccharomyces cerevisiae* and *Lachancea thermotolerans* during fermentation. Fermentation.

[46] Bagheri B., Bauer F.F., Cardinali G., Setati M.E. (2020). Ecological interactions are a primary driver of population dynamics in wine yeast microbiota during fermentation. Sci. Rep..

[47] Balsa-Canto E., Alonso-del-Real J., Querol A. (2020). Temperature shapes ecological dynamics in mixed culture fermentations driven by two species of the *Saccharomyces* genus. Front. Bioeng. Biotechnol..

