# Supplementary material for: Interactions of Saccharomyces cerevisiae and Lactiplantibacillus plantarum Isolated from Light-Flavor Jiupei at Various Fermentation Temperatures
Source: Foods. 2024 Sep 12;13(18):2884. doi: 10.3390/foods13182884 (PMC11431660; doi:10.3390/foods13182884)
Supplement: Supplementary file 1 [file foods-13-02884-s001.zip › TableS1 Biomass.pdf]

Table S1 T-test of microbial biomass between monoculture and coculture systems

	Saccharomyces cerevisiae			*Lactiplantibacillus plantarum*		
	Monoculture	Coculture	*P* value	Monoculture	Coculture	*P* value
30 °C 1:1						
0 h	5.65±0.09	5.92±0.04	0.0850	6.08±0.05	5.65±0.07	0.0262
6 h	6.75±0.11	6.52±0.07	0.2040	6.75±0.05	6.56±0.12	0.1866
12 h	7.52±0.13	7.34±0.14	0.3069	7.81±0.03	7.57±0.13	0.0912
Day1	7.73±0.13	7.68±0.04	0.7072	8.36±0.03	7.83±0.12	0.0331
Day2	7.70±0.08	7.52±0.05	0.0905	8.31±0.06	7.57±0.05	0.0002
Day4	7.75±0.07	7.65±0.08	0.4252	8.03±0.09	7.34±0.13	0.0116
Day7	7.69±0.09	7.63±0.07	0.6365	7.69±0.02	7.31±0.12	0.0393
Day10	7.33±0.06	6.96±0.15	0.0423	7.33±0.03	6.59±0.05	0.0040
27 °C 1:1						
0 h	5.94±0.02	5.98±0.09	0.6501	5.61±0.07	5.74±0.06	0.0361
6 h	6.54±0.05	6.42±0.12	0.1690	6.23±0.16	6.22±0.05	0.9835
12 h	7.23±0.07	7.09±0.05	0.1988	6.92±0.19	6.98±0.14	0.5997
Day1	7.59±0.10	7.70±0.06	0.4054	8.15±0.07	7.43±0.08	0.0084
Day2	7.62±0.06	7.58±0.04	0.6349	8.25±0.04	7.44±0.06	0.0080
Day4	7.42±0.11	7.54±0.14	0.1838	8.11±0.10	7.35±0.06	0.0037
Day7	7.60±0.12	7.79±0.11	0.0143	7.84±0.07	7.36±0.13	0.0791
Day10	7.69±0.10	7.11±0.12	0.0637	7.88±0.12	7.05±0.06	0.0198
24 °C 1:1						

0 h	6.00±0.09	6.07±0.04[D]	0.1902	5.55±0.07	5.69±0.05	0.2254
6 h	6.52±0.07	6.46±0.15	0.7686	5.82±0.11	5.68±0.00	0.2149
12 h	7.28±0.05	7.29±0.05	0.6710	6.86±0.12	6.86±0.11	0.9804
Day1	7.45±0.03	7.57±0.13	0.3502	8.33±0.07	7.64±0.13	0.0034
Day2	7.50±0.08	7.70±0.07	0.1756	8.35±0.03	7.70±0.10	0.0054
Day4	7.72±0.11	7.59±0.07	0.4157	8.33±0.03	7.55±0.03	0.0010
Day7	7.58±0.05	7.61±0.02	0.5380	8.32±0.06	7.46±0.07	0.0039
Day10	7.47±0.01	7.35±0.02	0.0104	7.90±0.09	7.24±0.18	0.0695
21 °C 1:1						
0 h	5.91±0.14	6.07±0.03	0.2556	6.07±0.04	5.93±0.02	0.0623
6 h	6.22±0.05	6.32±0.18	0.5589	6.31±0.01	6.08±0.04	0.0243
12 h	6.84±0.07	6.55±0.04	0.0557	6.59±0.10	6.46±0.20	0.2493
Day1	7.33±0.10	7.44±0.18	0.3708	6.76±0.02	7.11±0.09	0.0152
Day2	7.66±0.04	7.74±0.08	0.4771	8.31±0.07	7.94±0.06	0.0214
Day4	7.68±0.10	7.72±0.05	0.4320	8.05±0.05	7.86±0.11	0.2079
Day7	7.49±0.06	7.41±0.08	0.4874	7.91±0.03	7.70±0.04	0.0141
Day10	7.41±0.08	7.32±0.17	0.6592	7.84±0.12	7.51±0.05	0.1031
18 °C 1:1						
0 h	5.9±0.16	5.89±0.11	0.9470	5.82±0.03	5.61±0.02	0.0072
6 h	6.07±0.05	6.11±0.06	0.4951	5.90±0.06	5.89±0.09	0.9576
12 h	6.44±0.08	6.28±0.06	0.0090	6.53±0.07	6.40±0.07	0.2920
Day1	7.23±0.05	6.95±0.13	0.1284	7.79±0.06	7.62±0.14	0.1549

Day2	7.24±0.24	7.49±0.08	0.1766	8.31±0.07	7.74±0.14	0.0636
Day4	7.48±0.12	7.64±0.03	0.2181	8.58±0.02	8.16±0.05	0.0150
Day7	7.55±0.09	7.62±0.15	0.7036	8.29±0.05	8.05±0.09	0.1172
Day10	7.50±0.25	7.34±0.12	0.6023	8.26±0.05	7.87±0.09	0.0444
15 °C 1:1						
0 h	5.69±0.05	5.82±0.08	0.2748	5.52±0.09	5.51±0.08	0.7418
6 h	5.78±0.11	5.78±0.14	0.9831	5.79±0.42	5.85±0.09	0.8427
12 h	6.33±0.05	6.37±0.13	0.7462	6.08±0.07	5.94±0.12	0.2442
Day1	6.83±0.02	6.85±0.07	0.8213	6.85±0.07	6.93±0.26	0.7003
Day2	7.35±0.10	7.15±0.08	0.2061	7.93±0.10	7.81±0.07	0.1602
Day4	7.40±0.14	7.56±0.19	0.4710	8.55±0.03	8.18±0.05	0.0029
Day7	7.40±0.06	7.42±0.06	0.7951	8.42±0.09	8.09±0.07	0.0199
Day10	7.55±0.02	7.44±0.13	0.4362	8.40±0.04	8.10±0.07	0.0596
12 °C 1:1						
0 h	5.90±0.01	5.94±0.08	0.5803	5.86±0.10	5.66±0.13	0.3577
6 h	5.92±0.07	5.80±0.19	0.2778	5.85±0.08	5.71±0.32	0.4830
12 h	6.06±0.04	6.15±0.07	0.2311	5.86±0.05	5.75±0.08	0.3500
Day1	6.31±0.10	6.33±0.10	0.8791	5.91±0.04	5.90±0.07	0.8967
Day2	6.70±0.04	6.73±0.30	0.8932	6.43±0.16	6.55±0.04	0.4721
Day4	7.18±0.06	7.12±0.03	0.4128	7.06±0.05	6.96±0.07	0.1074
Day7	7.18±0.03	7.35±0.08[A]	0.0926	7.99±0.10	7.90±0.10	0.5044
Day10	7.27±0.03	7.19±0.04	0.0063	8.10±0.10	7.95±0.10	0.2800

9 °C 1:1						
0 h	5.86±0.16	5.84±0.04	0.8603	5.65±0.10	5.80±0.09	0.0148
6 h	6.04±0.02	5.80±0.05	0.0346	5.94±0.06	5.80±0.05	0.0171
12 h	5.91±0.03	6.03±0.07	0.1077	5.87±0.01	5.72±0.06	0.0998
Day1	6.23±0.02	6.15±0.03	0.1504	6.09±0.06	5.67±0.11	0.0555
Day2	6.53±0.10	6.52±0.16	0.7397	6.52±0.01	6.33±0.33	0.4992
Day4	7.23±0.08	7.10±0.07	0.2770	7.16±0.04	6.98±0.09	0.0725
Day7	7.20±0.08	7.30±0.03	0.3438	7.79±0.15	7.76±0.10	0.5352
Day10	7.18±0.04	7.16±0.05	0.7745	8.19±0.05	8.01±0.07	0.0977
30 °C 1:10						
0 h	5.96±0.20	5.99±0.07	0.7418	6.94±0.20	6.90±0.14	0.8551
6 h	6.91±0.15	6.98±0.10	0.3636	8.12±0.06	8.07±0.05	0.5194
Day1	7.70±0.03	7.46±0.03	0.0224	8.71±0.05	8.36±0.03	0.0123
Day2	7.68±0.04	7.62±0.09	0.3261	8.69±0.29	8.15±0.21	0.0785
Day4	7.72±0.11	7.39±0.18	0.0817	8.13±0.21	7.79±0.14	0.3047
Day7	7.50±0.09	7.19±0.11	0.0631	7.94±0.04	7.05±0.34	0.0740
Day10	7.61±0.06	6.50±0.14	0.0032	7.14±0.17	6.60±0.24	0.1245
21 °C 1:10						
0 h	5.95±0.07	6.06±0.03	0.0669	6.77±0.15	6.83±0.02	0.6476
6 h	6.52±0.03	6.40±0.07	0.2133	7.43±0.08	7.39±0.06	0.6501
Day1	7.51±0.04	7.48±0.07	0.7661	8.39±0.25	8.48±0.12	0.5352
Day2	7.62±0.09	7.35±0.20	0.1329	8.86±0.24	8.33±0.05	0.0904

Day4	7.69±0.10	7.54±0.17	0.2021	8.41±0.12	8.26±0.09	0.4417
Day7	7.90±0.07	7.59±0.18	0.1943	8.64±0.03	8.12±0.10	0.0143
Day10	7.65±0.02	7.23±0.23	0.1412	8.06±0.10	7.74±0.22	0.2428
15 °C 1:10						
0 h	5.99±0.07	5.89±0.02	0.2079	6.95±0.07	6.81±0.09	0.0708
6 h	6.25±0.09	6.30±0.04	0.3416	7.22±0.05	7.29±0.02	0.2767
Day1	6.98±0.09	6.94±0.10	0.8024	7.83±0.11	7.78±0.10	0.6723
Day2	7.76±0.13	7.45±0.28	0.3509	8.85±0.03	8.06±0.12	0.0167
Day4	7.46±0.12	7.36±0.06	0.2601	8.47±0.07	8.13±0.13	0.0679
Day7	7.46±0.04	7.34±0.18	0.5229	8.66±0.03	8.12±0.07	0.0071
Day10	7.61±0.02	7.42±0.02	0.0205	8.46±0.19	8.14±0.06	0.0915

Note: Data present as mean ± standard deviation.
