# Supplementary material for: Interactions of Saccharomyces cerevisiae and Lactiplantibacillus plantarum Isolated from Light-Flavor Jiupei at Various Fermentation Temperatures
Source: Foods. 2024 Sep 12;13(18):2884. doi: 10.3390/foods13182884 (PMC11431660; doi:10.3390/foods13182884)
Supplement: Supplementary file 1 [file foods-13-02884-s001.zip › TableS2 pH.pdf]

Table S2 One-way analysis of variance of pH between monoculture and coculture systems

	Saccharomyces cerevisiae monocultrure	Coculture	*Lactiplantibacillus plantarum* monoculture
30 °C 1:1			
0 h	5.39±0.01[b]	5.40±0.00[b]	5.43±0.01[a]
6 h	5.12±0.01[b]	5.09±0.00[c]	5.37±0.01[a]
12 h	4.71±0.02[a]	4.40±0.01[c]	4.59±0.03[b]
Day1	4.35±0.06[a]	3.96±0.02[b]	3.64±0.00[c]
Day2	4.03±0.07[a]	3.82±0.02[b]	3.39±0.00[c]
Day4	4.02±0.04[a]	3.76±0.00[b]	3.27±0.00[c]
Day7	4.00±0.04[a]	3.72±0.00[b]	3.22±0.01[c]
Day10	3.98±0.03[a]	3.71±0.01[b]	3.19±0.00[c]
27 °C 1:1			
0 h	5.40±0.01[a]	5.37±0.01[b]	5.41±0.00[a]
6 h	5.22±0.00[b]	5.17±0.01[c]	5.39±0.00[a]
12 h	4.82±0.02[a]	4.70±0.02[b]	5.19±0.03[a]
Day1	4.58±0.02[a]	4.19±0.05[a]	3.92±0.04[b]
Day2	4.36±0.02[a]	3.91±0.02[a]	3.45±0.02[b]
Day4	4.19±0.03[a]	3.77±0.02[b]	3.23±0.11[c]
Day7	4.24±0.04[a]	3.68±0.00[b]	3.21±0.02[c]
Day10	4.20±0.08[a]	3.66±0.02[b]	3.15±0.01[c]
24 °C 1:1			
0 h	5.46±0.01[a]	5.44±0.01[b]	5.47±0.01[a]

6 h	5.23±0.00^{b}	5.20±0.03^{b}	5.45±0.00^{a}
12 h	4.83±0.00^{b}	4.77±0.01^{b}	5.36±0.00^{a}
Day1	4.55±0.02^{a}	4.24±0.01^{b}	3.92±0.01^{c}
Day2	4.40±0.03^{a}	3.96±0.02^{b}	3.51±0.01^{c}
Day4	4.20±0.06^{a}	3.82±0.02^{b}	3.36±0.00^{c}
Day7	4.15±0.03^{a}	3.71±0.00^{b}	3.23±0.00^{c}
Day10	4.12±0.02^{a}	3.67±0.01^{b}	3.23±0.00^{c}
21 °C 1:1			
0 h	5.40±0.01^{a}	5.38±0.02^{a}	5.39±0.00^{a}
6 h	5.19±0.01^{b}	5.16±0.01^{b}	5.24±0.02^{a}
12 h	4.98±0.01^{a}	4.96±0.01^{b}	5.21±0.01^{b}
Day1	4.66±0.01^{b}	4.45±0.01^{c}	4.95±0.06^{a}
Day2	4.47±0.07^{a}	4.01±0.04^{b}	3.69±0.04^{c}
Day4	4.41±0.00^{a}	3.77±0.02^{b}	3.47±0.02^{c}
Day7	4.40±0.02^{a}	3.74±0.03^{b}	3.43±0.02^{c}
Day10	4.29±0.06^{a}	3.71±0.02^{b}	3.43±0.04^{c}
18 °C 1:1			
0 h	5.44±0.00^{b}	5.42±0.01^{b}	5.47±0.01^{a}
6 h	5.40±0.00^{b}	5.37±0.00^{c}	5.46±0.01^{a}
12 h	5.26±0.03^{b}	5.23±0.02^{b}	5.43±0.01^{a}
Day1	4.86±0.00^{b}	4.66±0.01^{c}	4.89±0.00^{a}
Day2	4.59±0.02^{a}	4.03±0.02^{b}	3.83±0.02^{c}

Day4	4.43±0.01[a]	3.74±0.01[b]	3.50±0.01[c]
Day7	4.15±0.06[a]	3.53±0.02[b]	3.28±0.00[c]
Day10	4.10±0.15[a]	3.44±0.01[b]	3.21±0.01[c]
15 °C 1:1			
0 h	5.45±0.00[b]	5.43±0.00[c]	5.47±0.00[a]
6 h	5.39±0.00[ab]	5.36±0.04[b]	5.45±0.00[a]
12 h	5.33±0.02[b]	5.30±0.00[c]	5.46±0.00[a]
Day1	5.07±0.02[b]	5.04±0.03[b]	5.35±0.00[a]
Day2	4.82±0.01[a]	4.46±0.02[c]	4.62±0.01[b]
Day4	4.45±0.01[a]	3.90±0.01[b]	3.69±0.00[c]
Day7	4.30±0.03[a]	3.63±0.01[b]	3.39±0.00[c]
Day10	3.96±0.06[a]	3.55±0.00[b]	3.31±0.05[c]
12 °C 1:1			
0 h	5.45±0.03[a]	5.41±0.01[a]	5.45±0.00[a]
6 h	5.42±0.01[b]	5.40±0.00[c]	5.45±0.00[a]
12 h	5.42±0.00[b]	5.40±0.01[c]	5.46±0.00[a]
Day1	5.35±0.01[b]	5.32±0.00[c]	5.46±0.00[a]
Day2	5.19±0.01[b]	5.12±0.02[c]	5.44±0.00[a]
Day4	4.88±0.01[b]	4.82±0.01[c]	5.26±0.00[a]
Day7	4.80±0.00[a]	4.27±0.02[c]	4.40±0.05[b]
Day10	4.70±0.06[a]	3.98±0.04[b]	3.89±0.02[b]
9 °C 1:1			

0 h	5.47±0.01^{a}	5.44±0.01^{b}	5.46±0.00^{ab}
6 h	5.46±0.01^{a}	5.42±0.01^{b}	5.46±0.01^{a}
12 h	5.45±0.01^{b}	5.40±0.00^{c}	5.48±0.01^{a}
Day1	5.38±0.01^{b}	5.36±0.01^{c}	5.46±0.00^{a}
Day2	5.22±0.01^{b}	5.17±0.00^{c}	5.44±0.00^{a}
Day4	4.87±0.01^{b}	4.78±0.00^{c}	5.16±0.03^{a}
Day7	4.78±0.03^{a}	4.34±0.07^{c}	4.54±0.04^{b}
Day10	4.72±0.00^{a}	4.04±0.00^{b}	3.98±0.02^{c}
30 °C 1:10			
0 h	5.53±0.01^{a}	5.37±0.01^{b}	5.35±0.00^{b}
6 h	5.27±0.01^{a}	4.75±0.01^{b}	4.79±0.03^{b}
Day1	4.62±0.01^{a}	3.77±0.02^{b}	3.59±0.02^{c}
Day2	4.48±0.05^{a}	3.67±0.01^{b}	3.49±0.00^{c}
Day4	4.30±0.03^{a}	3.56±0.03^{b}	3.34±0.02^{c}
Day7	4.37±0.03^{a}	3.57±0.04^{b}	3.27±0.00^{c}
Day10	4.25±0.03^{a}	3.48±0.00^{b}	3.15±0.01^{c}
21 °C 1:10			
0 h	5.51±0.01^{a}	5.39±0.01^{b}	5.37±0.01^{b}
6 h	5.46±0.00^{a}	5.19±0.00^{c}	5.21±0.00^{b}
Day1	4.82±0.01^{a}	4.05±0.02^{b}	3.98±0.01^{c}
Day2	4.66±0.05^{a}	3.91±0.00^{b}	3.69±0.01^{c}
Day4	4.55±0.09^{a}	3.66±0.02^{b}	3.51±0.00^{c}

Day7	4.35±0.03[a]	3.54±0.01[b]	3.35±0.02[c]
Day10	4.21±0.05[a]	3.41±0.01[b]	3.25±0.00[c]
15 °C 1:10			
0 h	5.49±0.01[a]	5.39±0.00[b]	5.37±0.00[c]
6 h	5.47±0.00[a]	5.24±0.01[c]	5.26±0.00[b]
Day1	5.19±0.01[a]	4.68±0.01[c]	4.84±0.01[b]
Day2	4.87±0.00[a]	4.12±0.01[c]	4.15±0.01[b]
Day4	4.80±0.01[a]	3.93±0.01[b]	3.81±0.01[c]
Day7	4.52±0.03[a]	3.61±0.16[b]	3.55±0.01[b]
Day10	4.51±0.04[a]	3.54±0.02[b]	3.44±0.01[c]

Note: Data present as mean ± standard deviation.
