# Supplementary material for: Interactions of Saccharomyces cerevisiae and Lactiplantibacillus plantarum Isolated from Light-Flavor Jiupei at Various Fermentation Temperatures
Source: Foods. 2024 Sep 12;13(18):2884. doi: 10.3390/foods13182884 (PMC11431660; doi:10.3390/foods13182884)
Supplement: Supplementary file 1 [file foods-13-02884-s001.zip › TableS3 Lactic acid.pdf]

Table S3 T-test of lactic acid yield between monoculture and coculture systems

	Lactiplantibacillus plantarum monoculture	Coculture	P value
30 °C 1:1			
0 h	0.00±0.00	0.00±0.00	1
6 h	0.00±0.00	0.00±0.00	1
12 h	0.81±0.06	0.78±0.06	0.0942
Day1	1.91±0.16	1.14±0.02	0.0151
Day2	2.98±0.41	1.47±0.06	0.0373
Day4	4.59±0.40	1.74±0.15	0.0179
Day7	5.29±0.14	2.30±0.20	0.0064
Day10	5.45±0.15	2.30±0.11	0.0017
27 °C 1:1			
0 h	0.00±0.00	0.00±0.00	1
6 h	0.00±0.00	0.00±0.00	1
12 h	0.39±0.02	0.10±0.01	0.0009
Day1	1.91±0.09	1.48±0.34	0.2262
Day2	2.71±0.07	1.69±0.38	0.0812
Day4	4.74±0.22	2.30±0.25	0.0086
Day7	4.88±0.40	2.95±0.28	0.0527
Day10	5.09±0.13	3.20±0.40	0.0360
24 °C 1:1			
0 h	0.00±0.00	0.00±0.00	1
6 h	0.00±0.00	0.00±0.00	1
12 h	0.26±0.07	0.13±0.02	0.1599
Day1	1.11±0.05	0.70±0.04	0.0165
Day2	3.33±0.35	1.18±0.04	0.0156
Day4	4.38±0.28	1.42±0.09	0.0039
Day7	4.72±0.06	1.92±0.17	0.0013
Day10	4.94±0.06	1.62±0.11	0.0012
21 °C 1:1			
0 h	0.00±0.00	0.00±0.00	1
6 h	0.00±0.00	0.00±0.00	1
12 h	0.24±0.02	0.03±0.00	0.0067
Day1	0.47±0.01	0.53±0.10	0.4846
Day2	2.07±0.20	1.23±0.10	0.0183
Day4	3.48±0.14	1.98±0.18	0.0200
Day7	4.03±0.10	2.53±0.16	0.0039

Day10	4.57±0.16	1.81±0.19	0.0055
18 °C 1:1			
0 h	0.00±0.00	0.00±0.00	1
6 h	0.00±0.00	0.00±0.00	1
12 h	0.23±0.02	0.03±0.00	0.0034
Day1	0.63±0.01	0.22±0.02	0.0018
Day2	1.64±0.09	0.90±0.02	0.0035
Day4	2.61±0.03	1.43±0.18	0.0141
Day7	3.60±0.13	1.85±0.14	0.0046
Day10	3.30±0.16	2.67±0.06	0.0255
15 °C 1:1			
0 h	0.00±0.00	0.00±0.00	1
6 h	0.00±0.00	0.00±0.00	1
12 h	0.27±0.02	0.02±0.00	0.0045
Day1	0.29±0.02	0.08±0.00	0.0017
Day2	0.75±0.04	0.73±0.06	0.7489
Day4	1.68±0.09	1.48±0.04	0.1511
Day7	2.55±0.09	1.83±0.07	0.0135
Day10	2.41±0.02	1.95±0.05	0.0028
12 °C 1:1			
0 h	0.00±0.00	0.00±0.00	1
6 h	0.00±0.00	0.00±0.00	1
12 h	0.03±0.04	0.00±0.00	1
Day1	0.43±0.41	0.05±0.01	0.3083
Day2	0.26±0.04	0.06±0.00	0.0185
Day4	0.31±0.02	0.09±0.00	0.0075
Day7	1.24±0.04	1.00±0.05	0.0469
Day10	0.78±0.06	0.58±0.03	0.0580
9 °C 1:1			
0 h	0.00±0.00	0.00±0.00	1
6 h	0.00±0.00	0.00±0.00	1
12 h	0.00±0.00	0.00±0.00	1
Day1	0.09±0.02	0.03±0.00	0.0416
Day2	0.21±0.01	0.05±0.00	0.0039
Day4	0.44±0.14	0.17±0.03	0.1329
Day7	0.78±0.28	0.91±0.11	0.5671
Day10	0.56±0.04	0.29±0.16	0.0887

30 °C 1:10			
0 h	0.00±0.00	0.00±0.00	1
6 h	0.00±0.00	0.00±0.00	1
Day1	2.10±0.09	1.61±0.01	0.0135
Day2	3.29±0.07	2.24±0.06	0.0017
Day4	4.30±0.04	2.59±0.16	0.0064
Day7	4.85±0.21	2.81±0.09	0.0091
Day10	5.09±0.05	2.88±0.05	9.15E-06
21 °C 1:10			
0 h	0.00±0.00	0.00±0.00	1
6 h	0.00±0.00	0.00±0.00	1
Day1	1.23±0.05	1.03±0.08	0.1438
Day2	2.20±0.04	1.53±0.03	0.0011
Day4	2.97±0.32	2.41±0.06	0.1354
Day7	4.01±0.12	2.65±0.12	0.0038
Day10	4.16±0.14	2.80±0.15	0.0038
15 °C 1:10			
0 h	0.00±0.00	0.00±0.00	1
6 h	0.00±0.00	0.00±0.00	1
Day1	0.33±0.01	0.42±0.08	0.2785
Day2	1.06±0.01	1.04±0.03	0.5101
Day4	1.80±0.04	1.50±0.04	0.0011
Day7	2.63±0.09	1.98±0.13	0.0275
Day10	2.94±0.10	2.49±0.01	0.0175

Note: Data present as mean ± standard deviation.
