# Supplementary material for: Interactions of Saccharomyces cerevisiae and Lactiplantibacillus plantarum Isolated from Light-Flavor Jiupei at Various Fermentation Temperatures
Source: Foods. 2024 Sep 12;13(18):2884. doi: 10.3390/foods13182884 (PMC11431660; doi:10.3390/foods13182884)
Supplement: Supplementary file 1 [file foods-13-02884-s001.zip › TableS4 Ethanol.pdf]

Table S4 T-test of ethanol yield between monoculture and coculture systems

	Saccharomyces cerevisiae monoculture	Coculture	*P* value
30 °C 1:1			
0 h	0.56±0.19	0.37±0.13	0.4634
6 h	1.00±0.17	0.89±0.08	0.3722
12 h	6.87±0.62	5.19±0.69	0.1335
Day1	9.32±1.31	8.70±1.67	0.6406
Day2	17.73±0.27	14.26±1.34	0.0562
Day4	27.90±0.94	26.16±2.28	0.3628
Day7	30.59±0.64	25.55±0.34	0.0103
Day10	19.18±1.24	21.77±1.14	0.1854
27 °C 1:1			
0 h	0.28±0.03	0.29±0.03	0.5799
6 h	0.73±0.02	0.80±0.05	0.3316
12 h	3.61±0.31	3.80±0.75	0.7645
Day1	12.39±0.54	12.55±1.19	0.9031
Day2	21.75±0.48	25.87±1.59	0.0986
Day4	42.51±2.34	38.52±1.11	0.2255
Day7	43.52±0.70	39.04±0.67	0.0153
Day10	32.18±0.64	34.07±2.39	0.3269
24 °C 1:1			
0 h	0.25±0.01	0.25±0.01	1.0000
6 h	0.75±0.02	0.68±0.10	0.4226
12 h	2.53±0.23	3.24±0.05	0.0454
Day1	8.33±0.79	8.83±0.46	0.1712
Day2	15.42±0.70	16.9±0.54	0.0380
Day4	20.85±0.98	26.97±1.00	0.0229
Day7	29.66±2.51	29.02±2.12	0.8541
Day10	27.61±1.39	24.61±1.48	0.0196
21 °C 1:1			
0 h	0.32±0.06	0.33±0.05	0.4226
6 h	0.48±0.05	0.61±0.15	0.4127
12 h	1.37±0.06	1.24±0.28	0.6323
Day1	6.79±0.56	7.07±0.96	0.8137
Day2	12.94±0.18	14.74±0.48	0.0140
Day4	27.38±0.50	22.31±1.19	0.0456
Day7	26.62±2.42	30.17±1.71	0.0503

Day10	21.29±1.40	20.34±0.59	0.5649
18 °C 1:1			
0 h	0.24±0.07	0.20±0.02	0.5874
6 h	0.38±0.12	0.29±0.01	0.3784
12 h	0.69±0.08	0.60±0.04	0.3455
Day1	2.82±0.31	2.59±0.13	0.5277
Day2	8.02±0.52	7.78±0.99	0.5511
Day4	16.31±0.26	17.21±0.64	0.1802
Day7	20.75±4.66	22.62±6.45	0.8180
Day10	16.66±1.25	13.58±0.89	0.0581
15 °C 1:1			
0 h	0.23±0.02	0.21±0.08	0.8000
6 h	0.25±0.01	0.25±0.01	1.0000
12 h	0.38±0.02	0.40±0.07	0.6349
Day1	1.02±0.08	1.36±0.47	0.4201
Day2	4.56±0.16	3.55±0.84	0.1781
Day4	9.37±0.83	11.56±0.21	0.0527
Day7	18.28±1.32	15.67±0.87	0.0147
Day10	27.33±1.13	29.82±0.25	0.0945
12 °C 1:1			
0 h	0.31±0.01	0.40±0.03	0.0741
6 h	0.32±0.02	0.42±0.04	0.0579
12 h	0.35±0.01	0.50±0.05	0.0454
Day1	0.56±0.05	0.66±0.03	0.1309
Day2	4.56±0.16	1.45±0.26	0.0086
Day4	12.03±2.03	11.58±0.21	0.7959
Day7	16.84±2.09	11.5±0.24	0.0766
Day10	7.34±0.42	7.95±1.95	0.6906
9 °C 1:1			
0 h	0.41±0.06	0.29±0.05	0.1617
6 h	0.47±0.05	0.47±0.11	0.9754
12 h	0.50±0.11	0.58±0.17	0.6684
Day1	0.65±0.14	0.57±0.06	0.2727
Day2	1.18±0.17	1.31±0.04	0.4513
Day4	5.68±1.48	4.30±1.05	0.2934
Day7	14.44±1.31	13.73±0.85	0.1638
Day10	8.83±1.21	9.00±1.07	0.2245

30 °C 1:10			
0 h	0.26±0.04	0.20±0.00	0.2020
6 h	1.15±0.12	1.12±0.11	0.8336
Day1	11.28±1.02	12.54±1.03	0.4475
Day2	24.14±1.62	24.19±2.39	0.9747
Day4	29.39±1.43	30.95±0.70	0.4072
Day7	31.26±0.91	29.55±0.52	0.0520
Day10	28.23±2.17	27.17±1.45	0.2698
21 °C 1:10			
0 h	0.26±0.01	0.23±0.01	0.1181
6 h	0.47±0.03	0.43±0.02	0.2293
Day1	5.88±0.13	5.10±0.25	0.0826
Day2	15.62±0.54	11.72±0.74	0.0178
Day4	24.75±0.36	22.37±1.99	0.2164
Day7	31.08±0.83	28.51±1.03	0.0067
Day10	29.57±0.94	24.82±1.7	0.1207
15 °C 1:10			
0 h	0.20±0.01	0.20±0.02	0.7892
6 h	0.34±0.02	0.45±0.06	0.1851
Day1	1.00±0.05	0.74±0.17	0.2339
Day2	4.82±0.38	5.73±0.56	0.2007
Day4	15.62±0.58	12.00±0.68	0.0037
Day7	16.72±2.55	21.62±1.45	0.0865
Day10	29.44±1.69	23.98±1.43	0.1240

Note: Data present as mean ± standard deviation.
