# Supplementary material for: Interactions of Saccharomyces cerevisiae and Lactiplantibacillus plantarum Isolated from Light-Flavor Jiupei at Various Fermentation Temperatures
Source: Foods. 2024 Sep 12;13(18):2884. doi: 10.3390/foods13182884 (PMC11431660; doi:10.3390/foods13182884)
Supplement: Supplementary file 1 [file foods-13-02884-s001.zip › TableS5 Reducing sugar.pdf]

Table S5 One-way analysis of variance of reducing sugar between monoculture and coculture systems

| | *Saccharomyces cerevisiae* monocultrure | Coculture | *Lactiplantibacillus plantarum* monoculture |
|---|---|---|---|
| 30 °C 1:1 | | | |
| 0 h | 49.13±1.49[a] | 46.94±1.39[ab] | 44.28±1.43[b] |
| 6 h | 45.68±1.18[b] | 48.87±1.31[a] | 43.55±0.90[b] |
| 12 h | 20.33±0.84[b] | 21.99±0.42[b] | 43.32±1.72[a] |
| Day1 | 18.27±0.83[c] | 21.45±0.42[b] | 43.94±1.05[a] |
| Day2 | 14.51±0.62[c] | 17.36±0.68[b] | 48.49±1.53[a] |
| Day4 | 1.98±0.13[b] | 2.21±0.08[b] | 44.33±0.90[a] |
| Day7 | 1.80±0.04[b] | 1.49±0.06[b] | 43.49±0.93[a] |
| Day10 | 1.79±0.18[b] | 1.67±0.13[b] | 43.28±1.29[a] |
| 27 °C 1:1 | | | |
| 0 h | 44.25±0.52[a] | 41.24±0.98[b] | 41.47±1.55[b] |
| 6 h | 42.22±1.23[a] | 40.23±0.91[a] | 40.86±0.98[a] |
| 12 h | 18.87±0.11[b] | 19.68±0.21[b] | 41.49±1.12[a] |
| Day1 | 17.76±0.35[b] | 18.79±0.80[b] | 41.50±0.73[a] |
| Day2 | 15.77±0.43[b] | 16.74±0.20[b] | 40.94±1.76[a] |
| Day4 | 1.91±0.08[b] | 2.92±0.16[b] | 38.69±0.81[a] |
| Day7 | 1.43±0.20[b] | 1.60±0.06[b] | 39.37±1.21[a] |
| Day10 | 1.79±0.05[b] | 2.04±0.09[b] | 38.39±0.73[a] |
| 24 °C 1:1 | | | |
| 0 h | 47.36±1.42[a] | 48.40±1.50[a] | 48.88±1.11[a] |

| | | | |
|---|---|---|---|
| 6 h | 46.55±1.01[a] | 48.60±1.64[a] | 48.51±1.44[a] |
| 12 h | 28.46±0.64[b] | 28.61±0.46[b] | 47.21±0.78[a] |
| Day1 | 24.66±0.79[b] | 25.72±0.45[b] | 47.36±0.98[a] |
| Day2 | 21.70±0.65[b] | 21.83±0.53[b] | 48.38±0.84[a] |
| Day4 | 9.83±0.10[b] | 10.15±0.38[b] | 49.63±0.80[a] |
| Day7 | 3.95±0.18[b] | 3.95±0.15[b] | 48.24±0.99[a] |
| Day10 | 3.19±0.12[b] | 3.32±0.16[b] | 49.02±1.37[a] |
| 21 °C 1:1 | | | |
| 0 h | 43.42±1.12[a] | 41.21±1.40[a] | 43.29±1.75[a] |
| 6 h | 37.08±1.29[b] | 38.77±1.36[b] | 43.46±1.25[a] |
| 12 h | 32.64±1.17[c] | 36.32±0.80[b] | 42.61±1.43[a] |
| Day1 | 29.34±0.67[c] | 32.91±0.81[b] | 42.46±1.86[a] |
| Day2 | 21.77±0.87[c] | 26.43±0.83[b] | 44.23±1.24[a] |
| Day4 | 9.36±0.23[b] | 11.35±1.26[b] | 40.74±0.84[a] |
| Day7 | 7.19±0.36[b] | 8.28±0.45[b] | 41.65±1.01[a] |
| Day10 | 2.22±0.08[b] | 2.44±0.14[b] | 40.84±1.20[a] |
| 18 °C 1:1 | | | |
| 0 h | 42.74±0.89[a] | 40.10±0.86[a] | 41.39±1.47[a] |
| 6 h | 40.16±0.87[a] | 39.74±1.05[a] | 39.72±1.45[a] |
| 12 h | 38.21±0.98[b] | 38.85±0.57[b] | 41.81±1.22[a] |
| Day1 | 35.11±1.55[b] | 36.18±1.42[b] | 40.08±0.92[a] |
| Day2 | 30.00±0.42[b] | 30.68±0.51[b] | 39.83±0.67[a] |

| | | | |
|---|---|---|---|
| Day4 | 19.40±0.19[b] | 18.78±0.31[b] | 39.54±0.53[a] |
| Day7 | 11.79±0.32[b] | 10.95±0.18[b] | 39.13±1.09[a] |
| Day10 | 4.49±0.18[b] | 4.59±0.19[b] | 39.92±0.68[a] |
| 15 °C 1:1 | | | |
| 0 h | 45.99±1.14[a] | 45.69±1.01[a] | 44.32±1.38[a] |
| 6 h | 46.70±1.14[a] | 44.50±0.86[a] | 44.74±0.67[a] |
| 12 h | 43.79±0.76[a] | 41.96±1.57[a] | 43.93±0.85[a] |
| Day1 | 39.17±1.05[b] | 36.18±0.92[c] | 46.39±1.17[a] |
| Day2 | 34.53±0.34[b] | 31.92±1.56[b] | 43.32±2.58[a] |
| Day4 | 17.11±1.06[c] | 20.68±0.43[b] | 45.09±0.51[a] |
| Day7 | 11.46±0.13[b] | 11.98±0.31[b] | 46.99±0.96[a] |
| Day10 | 4.19±0.12[b] | 4.15±0.19[b] | 45.36±1.23[a] |
| 12 °C 1:1 | | | |
| 0 h | 46.68±1.03[a] | 46.38±1.56[a] | 45.20±3.43[a] |
| 6 h | 47.15±0.44[a] | 45.96±1.14[a] | 47.60±0.87[a] |
| 12 h | 44.70±2.03[a] | 44.92±1.27[a] | 46.89±1.23[a] |
| Day1 | 46.41±1.37[a] | 45.66±1.11[a] | 48.18±0.69[a] |
| Day2 | 39.86±1.76[b] | 37.90±0.99[b] | 45.35±0.61[a] |
| Day4 | 24.72±0.78[b] | 24.50±0.81[b] | 46.25±1.36[a] |
| Day7 | 12.76±0.48[b] | 13.53±0.28[b] | 46.91±0.55[a] |
| Day10 | 11.32±0.36[c] | 13.26±0.37[b] | 44.63±0.63[a] |
| 9 °C 1:1 | | | |

| | | | |
|---|---|---|---|
| 0 h | 47.54±0.98[a] | 46.49±1.40[a] | 47.98±1.93[a] |
| 6 h | 47.06±0.59[a] | 43.69±1.30[b] | 44.66±1.19[ab] |
| 12 h | 47.52±1.78[a] | 47.47±1.90[a] | 46.48±1.37[a] |
| Day1 | 46.47±1.91[a] | 45.50±1.08[a] | 45.39±1.68[a] |
| Day2 | 41.67±1.08[b] | 42.71±1.71[ab] | 45.32±1.42[a] |
| Day4 | 35.20±1.54[b] | 36.15±1.00[b] | 47.01±1.09[a] |
| Day7 | 24.76±0.90[b] | 27.92±0.68[b] | 44.76±0.76[a] |
| Day10 | 16.64±0.59[b] | 19.02±0.53[b] | 44.53±1.63[a] |
| 30 °C 1:10 | | | |
| 0 h | 46.38±0.97[a] | 47.02±0.56[a] | 45.83±1.50[a] |
| 6 h | 41.52±0.80[b] | 45.36±0.70[a] | 44.86±1.48[a] |
| Day1 | 19.84±0.63[c] | 24.17±0.77[b] | 46.65±1.08[a] |
| Day2 | 14.11±0.29[c] | 17.46±0.56[b] | 45.84±1.20[a] |
| Day4 | 2.62±0.11[b] | 3.23±0.12[b] | 46.04±0.52[a] |
| Day7 | 1.32±0.09[b] | 1.33±0.09[b] | 46.18±1.16[a] |
| Day10 | 1.16±0.11[b] | 1.10±0.10[b] | 46.49±1.42[a] |
| 21 °C 1:10 | | | |
| 0 h | 44.72±1.06[a] | 43.90±1.35[a] | 44.14±1.64[a] |
| 6 h | 42.70±0.56[a] | 43.27±0.91[a] | 44.37±0.68[a] |
| Day1 | 34.24±0.91[c] | 36.13±0.35[b] | 43.25±0.54[a] |
| Day2 | 21.59±0.61[c] | 24.11±0.50[b] | 43.10±1.53[a] |
| Day4 | 10.63±0.62[b] | 11.89±0.62[b] | 42.39±1.16[a] |

| | | | |
|---|---|---|---|
| Day7 | 6.85±0.27$^{b}$ | 7.11±0.22$^{b}$ | 41.99±1.39$^{a}$ |
| Day10 | 3.03±0.27$^{b}$ | 3.46±0.10$^{b}$ | 43.79±1.46$^{a}$ |
| 15 °C 1:10 | | | |
| 0 h | 47.51±0.60$^{a}$ | 48.23±0.67$^{a}$ | 48.17±1.48$^{a}$ |
| 6 h | 48.13±1.29$^{a}$ | 48.59±1.00$^{a}$ | 47.09±0.66$^{a}$ |
| Day1 | 47.79±1.41$^{a}$ | 48.46±1.62$^{a}$ | 46.94±0.67$^{a}$ |
| Day2 | 35.41±0.84$^{c}$ | 38.72±1.12$^{b}$ | 47.89±1.38$^{a}$ |
| Day4 | 21.30±0.42$^{b}$ | 21.76±0.38$^{b}$ | 47.33±0.95$^{a}$ |
| Day7 | 10.97±0.32$^{b}$ | 11.28±0.50$^{b}$ | 46.51±1.25$^{a}$ |
| Day10 | 3.88±0.15$^{b}$ | 4.37±0.17$^{b}$ | 46.22±1.44$^{a}$ |

Note: Data present as mean ± standard deviation.
