# Supplementary figures and images for: Interactions of Saccharomyces cerevisiae and Lactiplantibacillus plantarum Isolated from Light-Flavor Jiupei at Various Fermentation Temperatures
Source: Foods. 2024 Sep 12;13(18):2884. doi: 10.3390/foods13182884 (PMC11431660; doi:10.3390/foods13182884)

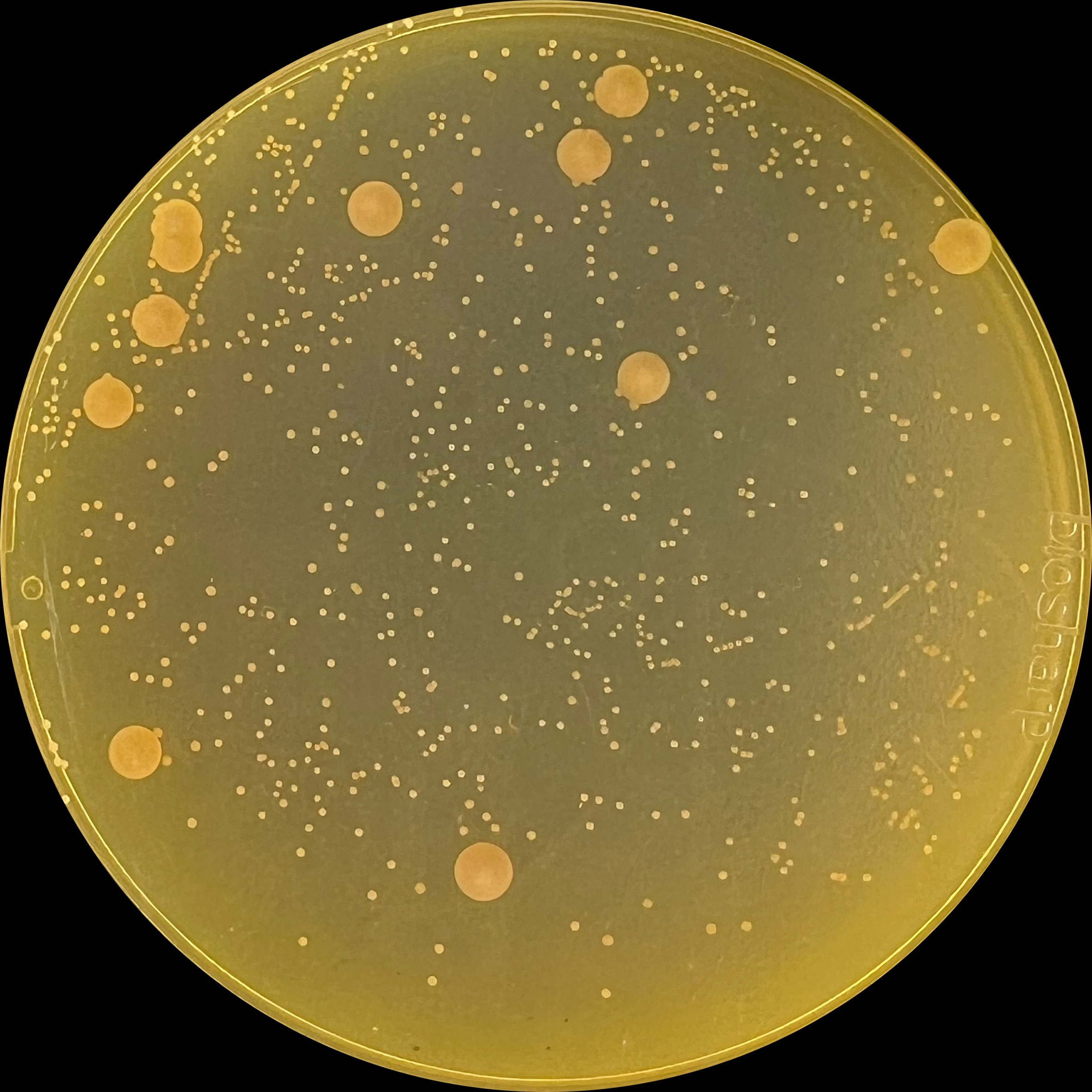

Supplement: Supplementary file 1 [file foods-13-02884-s001.zip › FigS1,colony morphology2.tif]

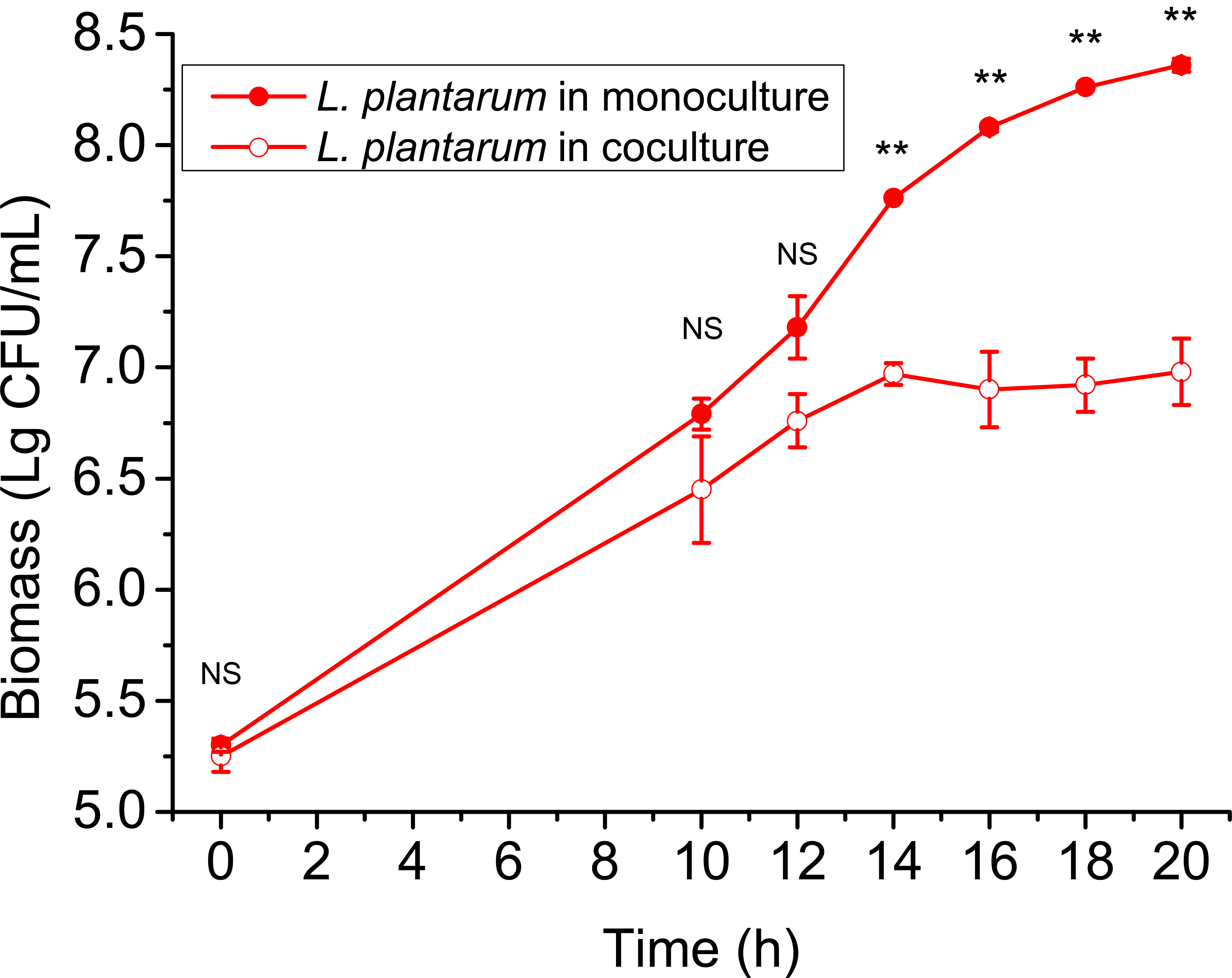

Supplement: Supplementary file 1 [file foods-13-02884-s001.zip › FigS2,Growth curve for proteomic.jpg]
